# Paralog‐Dependent Specialization of Paf1C Subunit, Ctr9, for Sex Chromosome Gene Regulation and Male Germline Differentiation in Drosophila

**DOI:** 10.1111/gtc.70040

**Published:** 2025-08-05

**Authors:** Toshie Kai, Jinglan Zheng, Taichiro Iki

**Affiliations:** ^1^ Laboratory of Germline Biology, Graduate School of Frontier Biosciences Osaka University Osaka Japan; ^2^ Graduate School of Biostudies Kyoto University Kyoto Japan

## Abstract

Testis‐specific gene regulatory mechanisms govern the differentiation of germ cells into mature sperm. However, the molecular underpinnings are not fully elucidated. Here, we show the subunits forming Paf1C, a transcription regulator complex conserved across eukaryotes, have their individual paralogs predominantly expressed in *Drosophila* testes. One of these, namely, Ctr9 paralog enriched in testes (Ctr9t) was found to play a critical and nonredundant role in postmeiotic spermatid differentiation and male fertility in *D. melanogaster* . A proximity proteome analysis provides evidence that Ctr9t prefers the interaction between paralog members. We show endogenous Ctr9t is expressed and functional in germ cells at spermatocyte stages, accumulating in a distinct compartment within the nucleolus. There, Ctr9t co‐localizes with Spermatocyte arrest (Sa), a testis‐specific paralog of TATA‐binding protein (TBP)‐associated factor 8 (TAF8). We further demonstrate that *ctr9t* function is crucial for maintaining Sa in the nucleolus, but not vice versa. Transcriptome profiling reveals that Ctr9t acts as an activator for the set of male fertility genes on the Y chromosome, but it also acts as a global repressor of X chromosome genes. Collectively, our results shed light on the nucleolus‐associated, paralog‐dependent regulation of gene expression from sex chromosomes, which ensures the terminal differentiation of male germ cells.

## Graphical abstract

We show that *Drosophila melanogaster* genome maintains testis‐specific paralogs encoding components of the transcription regulator Paf1C. One of the paralog products, Ctr9t, forms a unique compartment within the spermatocyte nucleolus. Ctr9t acts as a suppressor of X chromosome genes and an activator of Y chromosome fertility genes, thereby ensuring functional sperm production.

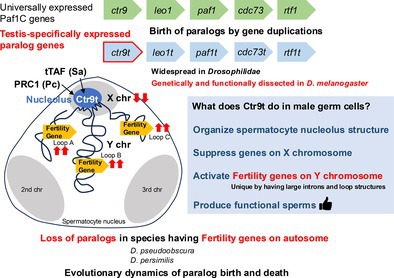

## 1 Introduction

Production of functional sperms is a prerequisite for the life cycles of sexually reproducing organisms. Spermatogenesis follows a multistep differentiation program, involving changes in cell cycle dynamics, morphogenesis, organella organization, and chromatin remodeling, generating a highly polarized haploid gamete having condensed DNA and a flagellum (Fabian and Brill 2012; Rathke et al. 2014). A wide repertoire of genes takes a coordinated action during these complex processes, the expression of which is controlled by diverse transcriptional and posttranscriptional mechanisms (Iki et al. 2023; Lim et al. 2012; Schä et al. 1995; White‐Cooper 2010). Hence, spermatogenesis provides an attractive model for elucidating the principle of tissue/cell‐specific gene regulation.

Gene expression programs in male germ cells require the functionally differentiated paralogs of general transcription regulators. In *Drosophila*, meiotic progression is preceded by transcriptional activation of an array of genes in premeiotic spermatocytes. This mass activation relies on two types of testis‐specific regulatory apparatuses. One is an alternative of TFIID, formed by testis‐specific TATA‐binding protein‐associated factors (tTAFs), Sa (dTAF8 paralog), Can (dTAF5 paralog), Rye (dTAF12 paralog), Mia (dTAF6 paralog), and Nht (dTAF4 paralog) (Chen et al. 2005; Hiller et al. 2004, 2001; Li et al. 2009; Lin et al. 1996; Metcalf and Wassarman 2007). The other module is meiosis arrest complex (tMAC), sharing a part of its subunits with the MMB/dREAM general repressor complex but replacing some subunits with testis‐specific homologs including Aly (Mip130 paralog), Tomb (Mip120 paralog), and Wuc (dLin52 paralog) (Beall et al. 2007; Doggett et al. 2011; Perezgasga et al. 2004). tMAC and tTAF cooperatively act over a thousand genes in spermatocytes, with the assistance of the Mediator complex and through the interdependency of subunit expression (Laktionov et al. 2018; Lu and Fuller 2015). Generally, the loss of either one of the above paralogs leads to the characteristic meiotic arrest phenotype during spermatogenesis, the failure of entry to meiotic divisions or initiation of spermatid differentiation, demonstrating their functions are nonredundant and distinct from those of universally expressed counterparts. Beyond their paramount importance for meiosis, the paralog activities are supportive of the postmeiotic terminal differentiation processes, given their responsibility for the expression of genes required at spermatid stages (Chen et al. 2005; Perezgasga et al. 2004).

Transcription initiation is followed by the sequence of events including RNA Polymerase II pausing, pause release, elongation, and termination, each of which is controlled by distinct sets of factors. One of the crucial modules controlling postinitiation steps is Polymerase associated factor 1 complex (Paf1C), originally identified in yeast with the five subunits, Paf1, Ctr9, Cdc73, Rtf1, and Leo1 (Francette et al. 2021; Jaehning 2010). These subunits are widespread across eukaryotes with minor differences in their composition; their general functions as gene activators have been demonstrated in different biological contexts. In *Drosophila*, Paf1/Antimeros binding to Cdc73/Hyrax is recruited to transcriptionally active loci, being required for H3K4 trimethylation (H3K4me3) and transcriptional activation upon heat shock (Adelman et al. 2006). Rtf1 colocalizes with Paf1 in transcriptionally active loci, though it lacks stable binding to Paf1. The role of Rtf1 in maintaining H3K4me3 levels has been shown in Notch signaling (Tenney et al. 2006). The gene activator function of Cdc73 has been demonstrated in Wnt signaling and the hedgehog pathway (Mosimann et al. 2006, 2009). The role of Ctr9 in maintaining proper H3K4me3 levels has been demonstrated in the nervous system and ovaries (Bahrampour and Thor 2016; Chaturvedi et al. 2016). A recent study characterized Paf1 and Rtf1 as antagonists of PIWI‐interacting (pi)RNA‐mediated gene silencing in ovarian somatic cell culture (Clark et al. 2017), which is analogous to the fission yeast Paf1C that opposes the short interfering (si)RNA pathway (Kowalik et al. 2015). Leo1 interacts with Myc and assists in binding to target gene promoters (Gerlach et al. 2017). Paf1C subunit activities in testes remain uncertain.

Here, we identify and characterize the paralogs of Paf1C subunits that are preferentially expressed in *Drosophila* testes. We generated a loss of function allele of one of those, Ctr9 paralog, demonstrating that the mutant is defective in spermatid differentiation and functional sperm production. By further combining proximity proteome, immunohistochemistry, and transcriptome data, our study suggests that Ctr9t paralog localizes to the spermatocyte nucleolus, where it interacts with other factors to regulate gene expression from sex chromosomes for maintaining male fertility.

## 2 Result

### 2.1 Drosophila Paralogs of Paf1C Subunits Are Predominantly Expressed in Testes

**FIGURE 1:**
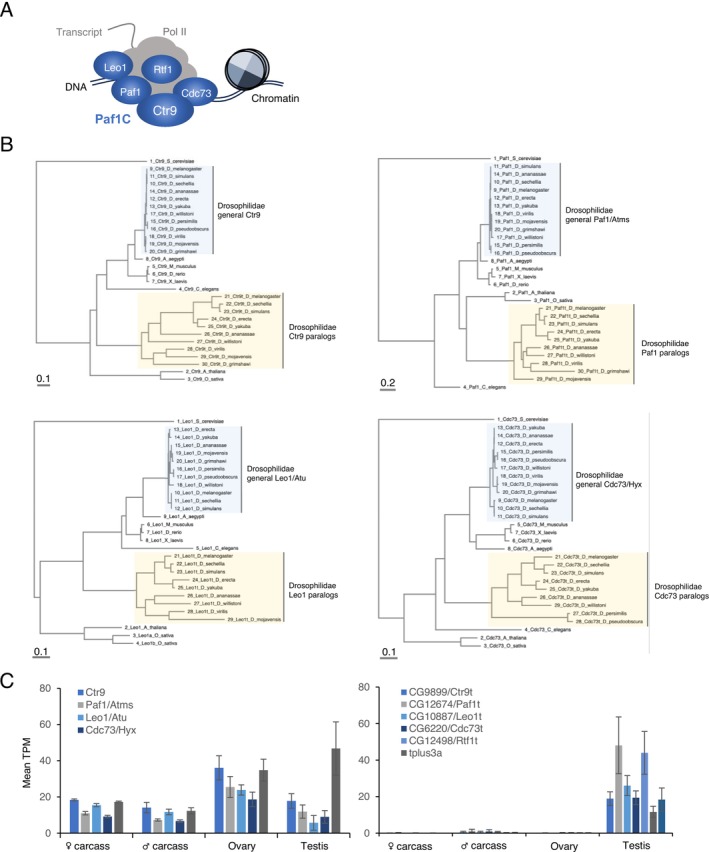
Paf1C subunit paralogs present in *Drosophilidae*. (A) Paf1 complex (Paf1C). Paf1C contains Ctr9, Paf1, Leo1, and Cdc73 as core components. Rtf1 is known to be dissociable. Paf1C interacts with RNA pol II and controls transcription‐associated events. (B) Multiple alignment and phylogenetic analyses of individual Paf1C subunit proteins. Branch lengths measure the expected substitutions per site, as indicated in the scale bar. (C) Expression patterns of genes encoding general Paf1C subunits (left) and their individual paralogs (right) in *D. melanogaster* . Source data: PRJEB22205. Mean TPM ± standard deviation of triplicate data is shown.

Paf1C is a complex conserved across eukaryotes, generally functioning as an activator of transcription through the control of associating RNA Polymerase II and the modulation of chromatin states (Francette et al. 2021). The core components, Paf1, Ctr9, Cdc73, Leo1, and the dissociable factor Rtf1, are widespread from yeast to humans (Figure 1A,B). Transcripts encoding the individual subunits, *Paf1/Atms*, *Ctr9*, *Leo1/Atu*, and *Cdc73/Hyx*, are expressed in different tissues of *D. melanogaster* , including ovaries and testes (Figure 1C, data publicly available as PRJEB22205 (Leader et al. 2018), see also FlyAtlas 2 https://motif.mvls.gla.ac.uk/FlyAtlas2/). Our BLAST search identified one paralog for each of Paf1/Atms, Ctr9, Leo1/Atu, and Cdc73/Hyx, respectively (Figure 1B). For Rtf1, three paralogs are found, of which tPlus3a and tPlus3b have been characterized previously as factors involved in spermatid differentiation (Figure S1A) (Hundertmark et al. 2019). Accumulating amino acid substitutions indicate the rapid evolution of paralogs compared to their individual counterparts (Figure 1B). These paralogs are widely conserved across *Drosophilidae*; however, for Paf1, Ctr9, and Leo1, we failed to identify their paralogs in *D. pseudoobscura* and the sympatric species, *D. persimilis* (Gao et al. 2007). On the other hand, a Cdc73 paralog can be found in the above two species, but not in *D. virilis* , *D. mojavensis* , and *D. grimshawi* .

We found that the paralog members are predominantly expressed in the testes of *D. melanogaster* , unlike their counterparts expressed universally (Figure 1C). Moreover, re‐analysis of the publicly available dataset, GSE99574 (Pal et al. 2023), revealed that the paralogs are testis‐specifically expressed in other species including *D. yakuba*, *D. ananasseae*, and *D. mojavensis* , suggesting that this expression pattern is conserved across *Drosophilidae* (Figure S1B). Based on their expression characteristics, the paralog genes are hereafter referred to as *ctr9* paralog enriched in testis (*ctr9t*)/*CG9899*, paf1 paralog enriched in testis (*paf1t*)/*CG12674*, leo1 paralog enriched in testis (*leo1t*)/*CG10887*, *cdc73* paralog enriched in testis (*cdc73t*)/*CG6220*, and rtf1 paralog enriched in testis (*rtf1t*)/*CG12498*, respectively.

### 2.2 *ctr9t* Is Crucial for Spermatid Differentiation and Male Fertility

**FIGURE 2:**
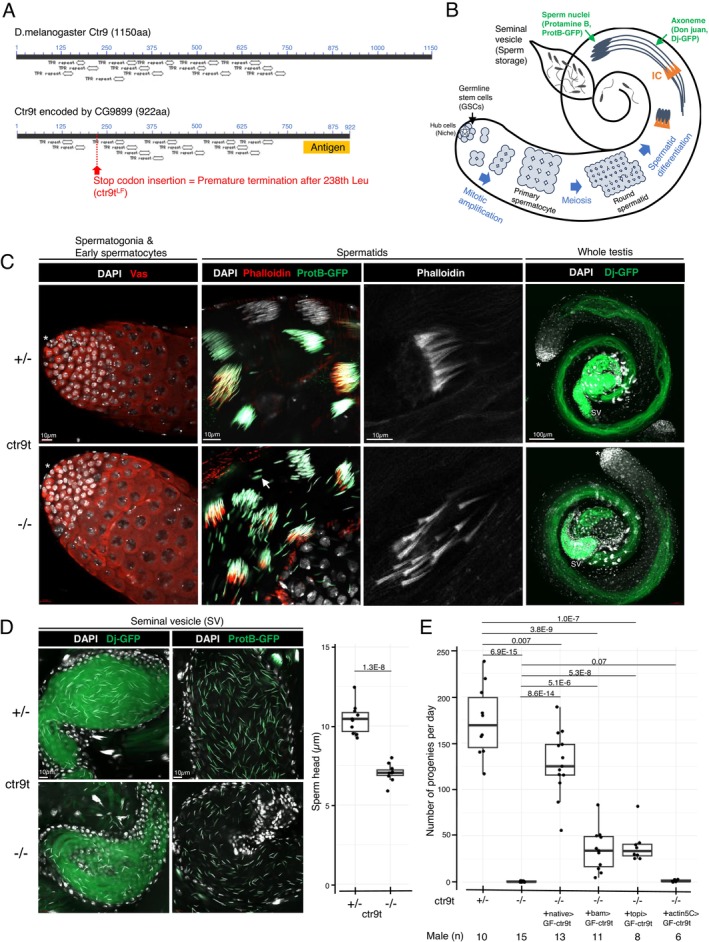
Genetic dissection of *ctr9t* function. (A) Polypeptide domain/motif analyses for Ctr9 and the paralogue Ctr9t. TPR; tetratricopeptide repeat. Images are taken from NCBI conserved domain platform. (B) Spermatogenesis in Drosophila testis. Note that somatic cells associated with germ cells are not described. IC; individualization complex. (C) Spermatogenesis in the presence (+/−) or absence (−/−) of *ctr9t*. DNA (DAPI), germ cells (Vas), IC (phalloidin), axoneme (Dj‐GFP), sperm nuclei (ProtB‐GFP). Asterisk, apical end of testis; SV, seminal vesicle. Arrow indicates abnormally dissociating nuclei. Magnified single IC shows its perturbation in the absence of *ctr9t*. (D) Magnified images for seminal vesicles and sperm stored inside. Length of sperm nuclei was measured and the result was summarized in the right panel. *p* value; two‐tailed unpaired *t*‐test. (E) Male fertility test. *p* values; two‐tailed unpaired *t*‐test.

Given the preferential expression in testes (Figure 1), the paralogs of Paf1C subunits might play a role in spermatogenesis. To examine this possibility, a loss‐of‐function allele of *ctr9t*/*CG9899* (*ctr9t* ^*LF*^) was generated in *D. melanogaster* using CRISPR‐Cas9‐based genome editing (Gokcezade et al. 2014). Ctr9 homologs share the characteristic tandem array of tetratricopeptide repeat (TPR) motifs involved in Paf1C formation (Francette et al. 2021). The introduced premature stop codon after 238th leucine was expected to truncate the polypeptide upstream of most TPR motifs maintained by Ctr9t (Figures 2A and S2A). In contrast to the null mutants of general *ctr9* that die before early larval stages (Bahrampour and Thor 2016; Chaturvedi et al. 2016), the homozygous *ctr9t* mutants (*ctr9t* ^*LF*/*LF*^) were viable and grew normally to adulthood. Immunoblotting with newly generated anti‐Ctr9t antibodies showed a distinct ~100 kDa band corresponding to the endogenous Ctr9t protein present in the lysates from *ctr9t* ^*LF*^/*CyO* heterozygous control, whereas the band was absent in *ctr9t* ^*LF*/*LF*^ mutant testes (Figure S2B).

An adult testis maintains germ cells at distinct differentiation stages in a spatially ordered manner, from germline stem cells at the apical end to maturing sperms in the basal end (Figure 2B). *ctr9t* mutant testes showed no discernible defect in spermatogonia and early spermatocytes (Figure 2C). However, at spermatid stages, elongating nuclei started to precociously dissociate, and characteristic F‐actin investment called the individualization complex (IC) became perturbed (Figure 2C). Nonetheless, spermatids developed flagellar axonemes marked by Don juan [Dj]‐GFP, and sperms accumulated protamines (ProtB‐GFP) in their nuclei and were stored in the seminal vesicles, similar to those in the control condition (Figure 2C,D). However, the stored sperm nuclei of *ctr9t* mutants were abnormally short (Figure 2D). Consistent with the defective morphology of sperms, *ctr9t* mutant males (*ctr9t* ^*LF*/*LF*^and *ctr9t* ^*LF*/*Df*^) were nearly sterile (Figures 2E and S2C). *ctr9t* was causal for these phenotypes, as sperm morphology and male fertility were recovered to levels nearly comparable to those of the control conditions by expressing GFP‐FLAG‐tagged Ctr9t under native promoter activity in *ctr9t* ^*LF*/*LF*^ mutants (Figures 2E and S2D). Accumulating GFP signals in testes implied Ctr9t functions in differentiating germ cells (Figure S2E). Indeed, those phenotypes showed weaker yet significant recovery by Gal4/UASp‐based *GFP‐FLAG‐ctr9t* expression using differentiating germline‐specific driver, *bam‐Gal4*, or spermatocyte‐specific driver, *topi‐Gal4* (Figures 2E and S2D,E) (Rørth 1998). The nonoptimal recovery could be due to the difference between native promoter and Gal4 driver activities; ectopic GFP signals were diffused in spermatogonia (*bam‐Gal4*) and late spermatocytes (*topi‐Gal4*), respectively (Figure S2E). In contrast to the germline drivers, no significant recovery was seen with a ubiquitous driver, *actin5C*‐*Gal4*, with which GFP signals were prematurely attenuated in spermatocytes (Figure S2E). Overall, these results indicate that Ctr9t is functionally distinct from general Ctr9 and plays a nonredundant role in germ cells for fertile sperm production.

Like general Ctr9 forming Paf1C, Ctr9t proteins may be able to form Paf1C‐like complexes with other paralog members in testicular germ cells. To examine this possibility, we analyzed the proximity proteome of Ctr9t by BioID/TurboID (Branon et al. 2018), expressing UASp‐*mini*(*m*)*Turbo‐FLAG‐ctr9t* using *bam‐Gal4*. The mass spectrometry did not provide sufficient peptide signals, probably due to the unstable nature and low steady‐state level of mTurbo‐FLAG‐Ctr9t proteins; but, nonetheless, Leo1t was repeatedly identified as the proximity factors of Ctr9t (Figure S2F,G). A parallel independent study has provided a deeper proximity dataset (Vilstrup et al. 2024). These results suggest that Ctr9t can form a complex with other testis‐specific paralog members in germ cells.

### 2.3 Ctr9t Localizes to a Compartment of Spermatocyte Nucleolus

**FIGURE 3:**
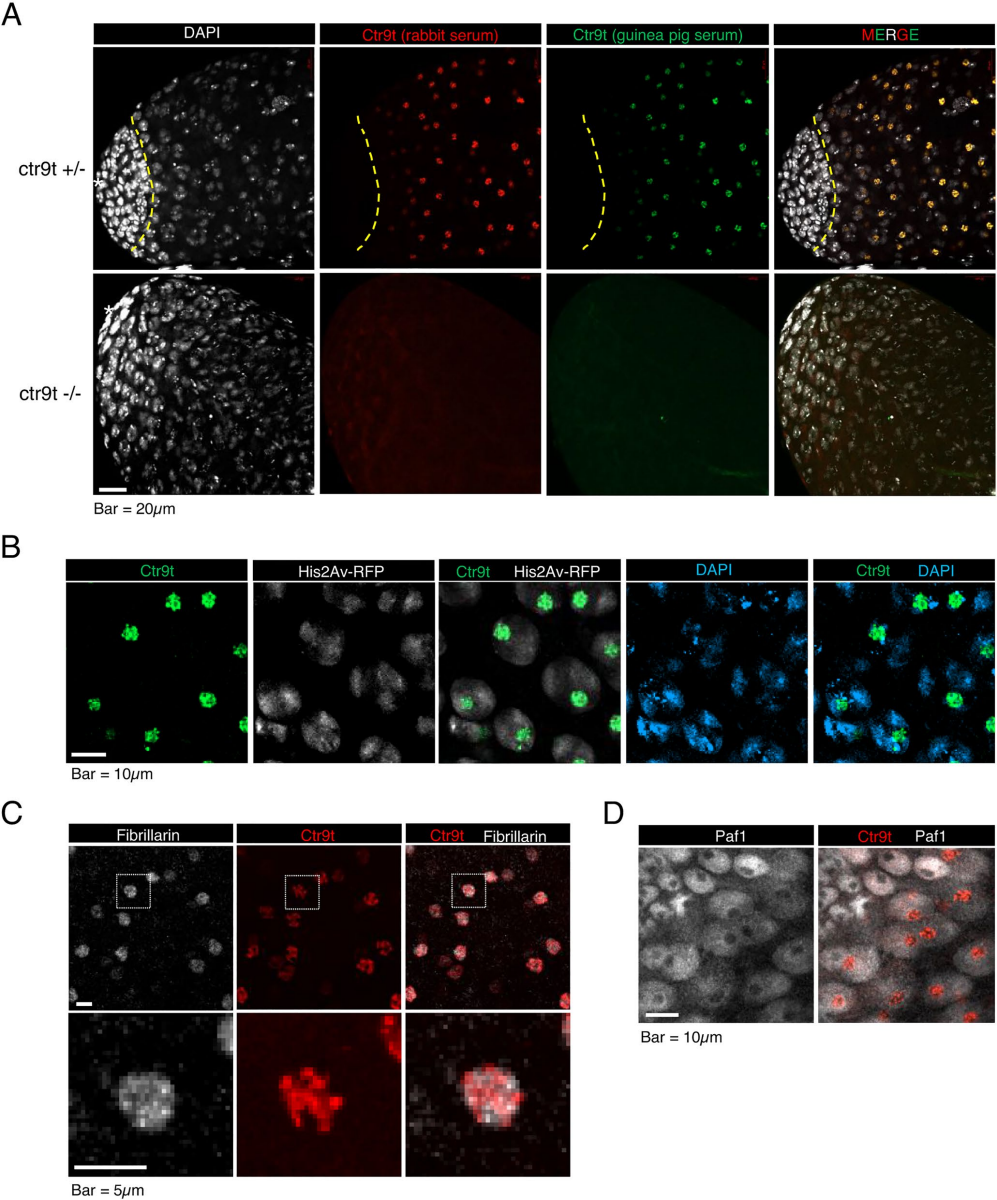
Ctr9t expression and localization in testes. (A) Testes immunostained for endogenous Ctr9t. Anti‐Ctr9t antibodies raised in rabbit (red) and guinea pig (green) showed identical signals in spermatocyte nuclei. Dotted yellow line roughly delineates the boundary between spermatogonia and spermatocytes. (B) Ctr9t (green), histone (H2Av‐RFP, white), and DNA (DAPI, blue) signals in spermatocyte nuclei. (C) Spermatocytes stained for Ctr9t (red) and a nucleolus marker, Fibrillarin (white). (D) Spermatogonia and spermatocytes immunostained for Paf1, a component of general Paf1C (gray), and Ctr9t (red).

To understand the molecular feature of Ctr9t, we next examined its expression and subcellular localization in testes. Immunostaining using anti‐Ctr9t antibodies showed that endogenous Ctr9t was enriched in a distinct structure in germ cells at spermatocyte stages (Figure 3A), in a pattern similar to GFP‐FLAG‐Ctr9t (Figure S2E). In contrast, Ctr9t was undetectable in spermatogonia, which was confirmed by the absence of Ctr9t signal in *bag‐of‐marbles* (*bam*) mutant testes where germ cell differentiation was arrested early at mitotic amplification steps (Figure S3A). Ctr9t signal partially overlapped with histone H2Av and DAPI signals, indicating that Ctr9t structure is formed inside the nucleus and associated with chromatin (Figure 3B).

Where within the nucleus does Ctr9t accumulate? One of the prominent subnuclear structures is the nucleolus, where various biological processes, including ribosome biogenesis, take place (Boisvert et al. 2007). Fibrillarin is a processing enzyme for ribosomal RNA and thus serves as a useful nucleolus marker. We found Ctr9t and Fibrillarin occupied the same region of the nucleus, though the two signals did not completely overlap (Figure 3C). This result suggests that Ctr9t is localized to the nucleolus of spermatocytes and concentrated in a specific compartment. Paf1 and Cdc73, the subunits of general Paf1C, accumulated in the nucleoplasm and did not co‐localize with Ctr9t (Figures 3D and S3B). In addition, H3K4me3, a histone mark associated with general Paf1C, was barely detectable in the nucleolar region (Figure S3C). These data support the notion that Ctr9t functions independently of general Paf1C.

### 2.4 Ctr9t Co‐Localizes With tTAF and PRC1 Components, and Controls Their Nucleolar Accumulation

**FIGURE 4:**
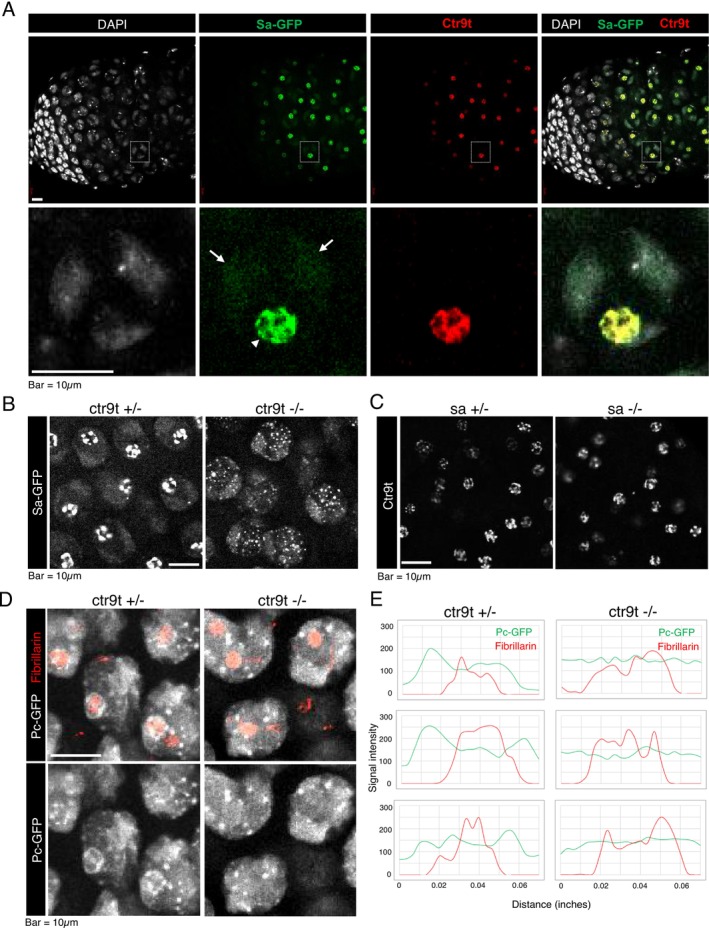
Co‐localization and dependency between Ctr9t and tTAF Sa. (A) Testes expressing Sa‐GFP (green) immunostained for Ctr9t (red). Magnified images of a spermatocyte nucleus (square box) are shown in the bottom panels. In addition to the signals on autosome regions (arrow), Sa‐GFP shows a strong signal enrichment in a nucleolar compartment where Ctr9t predominantly accumulates (arrowhead). (B) Effect of *ctr9t* mutation on the localization of Sa‐GFP in spermatocyte nuclei. (C) Effect of *sa* mutation on Ctr9t nucleolar localization. (D) Effect of *ctr9t* mutation on the localization of Pc‐GFP in spermatocyte nuclei. The yellow lines in the upper panels were used for GFP signal quantification in Panel E. (E) Quantification of nucleolar Pc‐GFP enrichment. Enrichment values were given by subtracting the basal signal from a measuring point outside of thenucleolus.

What could be the functions of Ctr9t enriched in a distinct compartment of the nucleolus? The characteristic localization of Ctr9t evoked that of tTAF Sa, an essential activator of genes involved in meiotic progression and postmeiotic differentiation of male germ cells (Chen et al. 2005). Sa has been reported to be enriched in spermatocyte nucleoli, exhibiting a pattern complementary to Fibrillarin, in addition to its localization to the condensing autosomes (Chen et al. 2005, 2011) (Figure 4A). Indeed, Ctr9t and Sa‐GFP showed a coincident expression pattern in testes, with their signals nearly perfectly overlapped in the spermatocyte nucleolus (Figure 4A). Strikingly, in the absence of *ctr9t*, Sa‐GFP failed to maintain the structure in the nucleolus, and instead formed smaller, abnormal speckles scattered throughout the nucleoplasm (Figure 4B). Sa‐GFP signal on autosomes, however, remained unaffected. In addition, Fibrillarin staining confirmed the nucleolus was maintained in *ctr9t* mutants (Figure S4A). The reciprocal analysis revealed that nucleolar localization of Ctr9t remained unchanged in the absence of *sa* (Figure 4C). One of the functions of tTAF Sa is to recruit the subunits forming Polycomb Repression Complex 1 (PRC1) including Polycomb (Pc) to the nucleolus (Chen et al. 2005). Consistent with the previous study, a fraction of Pc‐GFP was enriched in and around the early spermatocyte nucleolus, where we found it partially co‐localized with Ctr9t (Figure S4B). In *ctr9t* mutants, the nucleolar periphery enrichment of Pc‐GFP was substantially decreased (Figure 4D,E). Taken together, these results indicate the *ctr9t*‐dependent nucleolar enrichment of tTAF Sa and PRC1 component Pc, raising a possibility of functional interaction between Ctr9t, Sa, and Pc in the spermatocyte nucleolus.

### 2.5 Ctr9t Regulates Gene Expression From Sex Chromosomes

**FIGURE 5:**
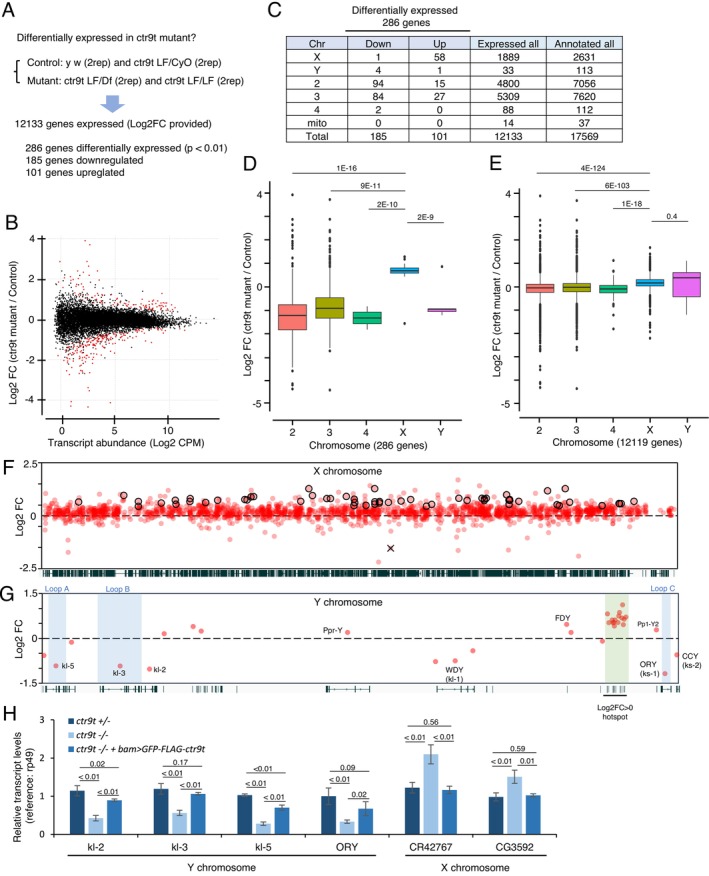
Effect of *ctr9t* mutation on transcript abundance in testes. (A) Analysis scheme of transcriptome sequencing data. Control and mutant conditions contain 4 data points (2 from *y w* and 2 from *ctr9t* ^*LF*/*CyO*^) and 4 data points (2 from *ctr9t* ^*LF*/*LF*^ and 2 from *ctr9t* ^*LF*/*Df*^), respectively. (B) Volcano plot shows the result of differential expression analysis using EdgeR. Red dot: 286 genes (exact *p* < 0.01). (C) Table for 286 genes differentially expressed in *ctr9t* mutants. Genes were grouped by their locating chromosome and transcript changing patterns (upregulated or downregulated in mutants). (D) Box plot for fold changes of transcript levels with *p* values (two‐tailed unpaired *t*‐test). Differentially expressed 286 genes are grouped by their locating chromosome. (E) A similar box plot covering all genes extracted from differential expression analysis (*n* = 12, 119). (F, G) Chromosome‐wide landscape of *ctr9t* mutation effect on transcript levels. Significantly upregulated 58 and downregulated 1 genes (exact *p* < 0.01, see Panel C) on X chr were highlighted with a circle and a cross, respectively. (H) RT‐qPCR measurement of transcript levels for selected genes on sex chromosomes. Four biological replicates. *p* values: two‐tailed unpaired *t*‐test.

**FIGURE 6:**
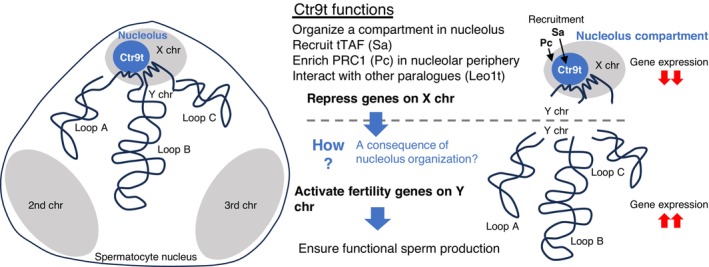
A model for the role of Ctr9t in spermatogenesis. Left: A schematic drawing of *D. melanogaster* spermatocyte nucleus. The condensed X chromosome is universally near the nucleolus. Y loops are formed in the nucleoplasm and thus are distant from the nucleolus. Right: A model summarizing the functions of Ctr9t. Ctr9t is exclusively enriched in the spermatocyte nucleolus, required for recruiting tTAF Sa and PRC1 component Pc. Interacting with these and potentially other factors, including testis‐specific paralogs of Paf1C, Ctr9t regulates gene expression from sex chromosomes. Ctr9t thereby ensures spermatid differentiation and male fertility.

Germ cells lacking *sa* do not undergo meiosis in testes (Lin et al. 1996), whereas those lacking *ctr9t* show clear defects in postmeiotic spermatid differentiation (Figure 2). This suggests the absolute necessity of tTAF Sa, but not of Ctr9t, for meiosis progression. Hence, although Ctr9t is important for maintaining Sa in the nucleolus (Figure 4), it is unlikely that Ctr9t is associated with the overall activities of Sa. To explore whether and how Ctr9t acts as a regulator of gene expression, we performed whole testicular transcriptome sequencing, compared the steady‐state transcript levels between controls (*LF*/*CyO*, *y w*) and *ctr9t* mutants (*LF*/*LF*, *LF*/*Df*), and identified 286 differentially expressed genes in the mutants (*p* < 0.01) (Figure 5A–C; Table S1). Among those, genes regulated by tTAF Sa, such as *mst87F*, *dj*, and *fzo* (Chen et al. 2011), were not included, and their transcript levels did not change dramatically in *ctr9t* mutants (Figure S5A). Remarkably, of 286 genes, downregulated 185 members contained *kl‐2*, *kl‐3*, *kl‐5*, and *ORY* (*ks‐1*) on the Y chromosome, all of which are male fertility genes crucial for spermatid differentiation (Figures 5C and S5B) (Hafezi et al. 2020; Zhang et al. 2020). The concomitant downregulation of the above 4 genes was intriguing given that the Y chromosome has only 14 confirmed protein‐coding genes (23 including predicted ones among 113 genes totally annotated) (Chang and Larracuente 2019; Kotov et al. 2022). Our data also highlighted that genes on the X chromosome occupy more than half of the upregulated members (58 out of 101), whereas only 1 on the X was found among the downregulated genes (1 out of 185, Figure 5C,D). A broader analysis of all genes (those given transcript fold changes [FC] in differential expression analysis) revealed widespread gene activation/derepression across the X chromosome upon *ctr9t* loss (Figures 5E,F and S5C). This was further confirmed by the re‐analysis of transcriptome data of whole testes or sorted spermatocytes lacking *ctr9L*(*ctr9t*) or the possible cofactor, *leo1L*(*leo1t*), reported by a parallel independent study (Figures S2G and S5D) (Vilstrup et al. 2024). Our in‐house transcriptome profiling identified 33 genes expressed from the Y chromosome, with the loss of *ctr9t* altering their expression levels in various ways (Figure 5E). Of those, 15 activated 15 genes showing log_2_FC > 0 were located within a specific region (Y:3,258,406‐3,365,197), implying a topological effect by *ctr9t* loss (Figures 5G and S5E). Outside of this narrow region, two additional fertility genes, *WDY* (*kl*‐*1*) and *CCY* (*ks*‐*2*) (Hafezi et al. 2020, 2024; Zhang et al. 2020), showed −0.75 and −0.56 of log_2_FC, respectively, and were grouped with *kl*‐*2*, *kl*‐*3*, *kl*‐*5*, and *ORY* as *ctr9t*‐dependent members (Figures 5G and S5E). In contrast, others dispensable for male fertility (*FDY*, *Ppr‐Y*, and *Pp1‐Y2*) (Hafezi et al. 2020; Zhang et al. 2020) did not rely on *ctr9t* (log_2_FC > 0). Therefore, on the Y chromosome, genes required for male fertility formed a tight association with *ctr9t* functions. These transcriptome results were confirmed by RT‐qPCR‐based measurement (Figures 5H and S5F). Of note, the transgene (*GFP‐FLAG‐ctr9t*) introduced in *ctr9t* mutants restored the nearly normal expression levels of misregulated genes on the X and Y chromosomes (Figures 5H and S5F). Altogether, these results highlight the unique regulatory roles of Ctr9t for gene expression from sex chromosomes (Figure 6).

## 3 Discussion

Tissue‐specific gene regulatory mechanisms can be established by acquiring functionally differentiated paralogs of general transcriptional regulators. This study extended this notion by identifying and functionally characterizing Ctr9t, the testis‐specific paralog of Ctr9. Proximity proteome profiling suggests that Ctr9t can act by forming a complex with other paralogs of individual subunits in Paf1C (Figure S2). Nonetheless, further genetic and functional dissection of Paf1t, Cdc73t, Leo1t, and Rtf1t is necessary to address the possibility. How have Paf1C paralogs been established during evolution? A molecular evolutionary study on tTAFs has shown that individual members arose by independent gene duplication events and evolved rapidly to acquire testis‐specific activities through reduced purifying selection, pervasive positive selection, and coevolution (Li et al. 2009). The above evolutionary study suggested that the scenario may apply to other genes displaying rapid evolution in *Drosophila* and characterized by testis‐enriched expression. Based on the phylogenetic data and expression profiles (Figures 1 and S1), we assume that duplication events occurred early in the ancestors of modern *Drosophila* species and have contributed to the acquisition of testis‐specific paralogs for Paf1C subunits.

Ctr9t is predominantly enriched in a compartment of spermatocyte nucleolus (Figure 3). There, Ctr9t co‐localizes almost perfectly with a tTAF member, Sa. In addition, nucleolus Sa localization requires *ctr9t* function (Figure 4). Considering these observations, we hypothesized that Ctr9t is involved in tTAF‐mediated events. However, unlike *sa* mutant germ cells which fail to undergo meiosis, germ cells lacking *ctr9t* can complete meiosis and progress to the spermatid stages (Figure 2). Moreover, expression of known tTAF targets was not dramatically affected in *ctr9t* mutants (Figure S5). Hence, it would be plausible to conclude that Ctr9t is not associated with overall tTAF activities. In agreement with this, the population of Sa localized in the autosome territories, which is capable of directly controlling tTAF targets, remained unaffected in *ctr9t* mutants (Chen et al. 2005, 2011) (Figure 4). What, then, could be the function(s) of Sa in the nucleolus? Perturbation of Sa enrichment in the nucleolus has been reported also in the mutant testes lacking THOC5, a component of THO complex mediating mRNA export (Moon et al. 2011). Similar to *sa* mutant, *THOC5* mutant testes show meiotic arrest phenotype. These pieces of evidence highlight the ability of *ctr9t* mutant germ cells to enter postmeiotic stages. Given the fact that nucleolus Sa disruption can be uncoupled from meiotic arrest, potential function(s) of nucleolar Sa may not be necessary for meiosis but rather important for postmeiotic differentiation.

Male fertility genes on the Y chromosome, including *kl‐2*, *kl‐3*, *kl‐5*, and *ORY* (*ks‐1*), depend on Ctr9t function for their expression (Figure 5). These fertility genes are required for spermatid differentiation, including the nuclear elongation (Hafezi et al. 2020; Zhang et al. 2020), like *ctr9t*, and we assume the activator role of Ctr9t is relevant for its importance in fertile sperm formation (Figure 2). All of these genes are characterized by containing gigantic introns, and accordingly, *kl‐3*, *kl‐5*, and *ORY* are known to form highly structured co‐transcriptional architecture called Y loops in the spermatocyte nuclei (Bonaccorsi et al. 1988; Fingerhut et al. 2019; Heatwole and Haynes 1996; Redhouse et al. 2011; Zhu and Fukunaga 2021). Thus, it remains unclear how Ctr9t, which is predominantly enriched in the nucleolus, can activate genes forming Y loops and being distant from the nucleolus (Figure 6). It was recently reported that the Y chromosome in spermatocytes is uniquely covered by H2A mono‐ubiquitinylation, a histone mark associated with polycomb‐mediated silencing in somatic cells (Aloia et al. 2013; Anderson et al. 2023). Following this, and based on the model originally proposed for the role of tTAF Sa (Chen et al. 2005), Ctr9t‐dependent sequestration of Pc (Figure 4) may reduce the access of polycomb to Y loops and prevent undesired silencing of fertility genes. From an evolutionary viewpoint, the set of male fertility genes is maintained by autosomes but not by the Y chromosome in *D. pseudoobscura*, due to the Y‐to‐dot translocation event that occurred 10–20 million years ago (Chang and Larracuente 2017; Larracuente and Clark 2014). In this regard, the absence of paralogs for *ctr9*, *paf1*, and *leo1* in *D. pseudoobscura* and closely related *D. persimilis* (Figure 1) might imply the particular importance of paralog functions for the expression of fertility genes from Y loops.

In addition to the role of Ctr9t in activating the fertility genes on the Y chromosome, our transcriptome analyses suggest that Ctr9t acts as a repressor of many genes on the X chromosome (Figure 5). Sex chromosome silencing during male meiosis has been observed across animals, though the mechanisms can differ (Hense et al. 2007; Kemkemer et al. 2011; Lifschytz and Lindsley 1972; Mahadevaraju et al. 2021; Meiklejohn et al. 2011; Vibranovski et al. 2009; Witt et al. 2021). In mammals, synapsis and recombination occur in autosomes but not in sex chromosomes, which initiates the condensation and transcriptional inactivation for maintaining genomic integrity (Abe et al. 2022; Lu and Yu 2015). However, *Drosophila* males lack synapsis and meiotic recombination even in autosomes, and hence, the selective inactivation of the X chromosome could have distinct biological relevance. Furthermore, the mechanisms underlying X inactivation in *Drosophila* remain largely unclear. The inactivation seems to far exceed the simple lack of the canonical dosage compensation mechanism (activation of single X) in testes (Hense et al. 2007; Mahadevaraju et al. 2021; Meiklejohn et al. 2011). Repressive histone modifications are not enriched in the X chromosome of spermatocytes (Anderson et al. 2023). These lines of evidence imply unrecognized, possibly *Drosophila*‐specific, molecular underpinnings of X chromosome inactivation. Ctr9t, the candidate factor identified in this study, is predominantly enriched in the nucleolus, which is universally adjacent to the condensed X chromosome in primary spermatocytes (Figure 3) (Mahadevaraju et al. 2021). Given that nucleoli play a key role in X chromosome inactivation in mammalian female cells (Zhang et al. 2007), it is plausible that Ctr9t may mediate a similar process in *Drosophila*. Ctr9t could exert its suppressor effect with other Paf1C paralogues. Indeed, loss of *leo1t* encoding the potential interactor of Ctr9t (Figure S2G) caused similar X chromosome‐wide gene derepression (Figure S5D). Moreover, of 58 genes significantly derepressed in *ctr9t* mutants (Figure 5B), 54 were also derepressed in the *leo1t* mutant (Table S1). In this regard, the mode of action of testis‐specific complexes containing Ctr9t would be dissimilar to that of Paf1C containing Ctr9, given that these universally expressed complexes generally act as transcription activators (Francette et al. 2021). However, it is important to note that the whole testis transcriptome profiling cannot rule out indirect effects caused by the loss of *ctr9t*. For example, loss of *ctr9t* led to the formation of small Sa speckles (Figure 4), which may have induced ectopic gain‐of‐function effects on gene expression. Although developmental defects may also affect the transcriptome, this appears unlikely in the *ctr9t* mutant, as the germ cells did not exhibit a strong arrest phenotype throughout the differentiation process (Figure 2). Future studies are needed to determine whether and how Ctr9t interacts with X chromosome genes.

Nucleolus acts as a central hub in mediating multiple events including ribosome assembly, DNA damage repair, stress response, apoptosis, and chromatin regulation (Boisvert et al. 2007; Feng and Manley 2022; Iarovaia et al. 2019). Recent studies highlight the interaction of the nucleolus with repressive chromatin domains (Bersaglieri et al. 2022; Peng et al. 2023). In *Drosophila* spermatocytes, proteins forming Polycomb repressive complex 1 (PRC1) including Pc can have a distinct role in the nucleolus, not being sequestered in an inactive state (Chen et al. 2005, 2011; El‐Sharnouby et al. 2013). Ctr9t‐dependently enriched Pc may contribute to the repressive activities of Ctr9t on the X chromosome (Figure 4). On the other hand, the nucleolar region contains elongating RNA Polymerase II and TAF1 (El‐Sharnouby et al. 2013; Metcalf and Wassarman 2007). In addition, acetylated histones and their testis‐specific reader proteins are also enriched in the nucleolus and partially co‐localize with Sa and Pc proteins (Kimura and Loppin 2015; Leser et al. 2012; Theofel et al. 2014, 2017). Hence, the spermatocyte nucleolus may exert differential effects on gene expression. Further study on Ctr9t and potentially functional interactors including tTAF Sa and Polycomb (Pc) could provide the mechanistic framework for understanding the gene regulatory activities of spermatocyte nucleoli.

Cytoplasmic dispersion of ectopically expressed Ctr9t (Figure S2) raises a question of what drives the enrichment in the nucleolus. General Ctr9 can be present in the cytoplasm when not forming Paf1C (De Gois et al. 2015). It is possible that the interaction between paralogs of Paf1C subunits enables the nucleolar localization. To support this, one of the Rtf1 paralogs, tPlus3b, that activates *kl‐3* and *kl‐5* genes similar to Ctr9t, can be found in the nucleolus (Hundertmark et al. 2019). Together with previous studies, our work proposes a model whereby different testis‐specific gene regulators functionally interact in the nucleolus. Links between paralogs would be of importance in this context. Further studies on *Drosophila* spermatogenesis will reveal otherwise unanticipated functions of the nucleolus in gene regulation.

## 4 Experimental Procedures

### 4.1 Fly Stocks and Cultures

Fly stocks are reared at 25°C on a molasses/yeast medium [5% (w/v) dry yeast, 5% (w/v) corn flower, 2% (w/v) rice bran, 10% (w/v) glucose, 0.7% (w/v) agar, 0.2% (v/v) propionic acid, and 0.05% (v/v) p‐hydroxy butyl benzoic acid]. The following stocks were used: *yellow white* (*y w*), *nos‐phiC31*; P{CaryP}attP2 (BL#25710), *nos‐phiC31*; PBac{y[+]‐attP‐3B}VK00033 (BL#32542), Df(2R)Exel7177/CyO (BL#7906, deficiency for *ctr9t*), *bam‐GAL4/TM6*, *topi‐GAL4/TM3* (BL#91776), *actin5C‐GAL4/TM6b* (BL#3954), *His2Av‐mRFP* (BL#23650), *bam* ^*Δ86*^/TM3 (BL#5427), *sa* ^2^/TM6B (Lin et al. 1996), *Sa‐GFP* (Chen et al. 2005), *Dj‐GFP*/CyO (BL#5417), *ProtB‐GFP*/CyO (DGRC#109173), *Pc‐GFP* (BL#9593).

### 4.2 Plasmid Construction, Fly Transformation, Mutagenesis

All the primers used for plasmid construction are listed in Table S2. For the establishment of *CG9899/ctr9t* ^*LF*^ by CRISPR‐Cas9, the left and right homology arms were amplified by pfusion PCR (NEB) using *y w* genomic DNA as the template and primer pairs Ti897/898 and Ti899/900. A fragment containing the *3xP3‐dsRed* marker was amplified using primer pairs Ti673/Mt024 (template: pBlue‐cyp40KOarm (Iki et al. 2020)). These three fragments were cloned into EcoRI‐digested pBluescript‐II vector using the In‐Fusion HD cloning kit (Takara). gRNA plasmids were constructed by cloning annealed oligo DNA (Ti902/903 and Ti904/905) into BbsI‐digested pDCC6 vector (Gokcezade et al. 2014). The homology arm plasmid and gRNA plasmids were co‐injected into *y w* embryos. For UASp‐*GFP‐3FLAG‐ctr9t*, *GFP‐3FLAG* and *ctr9t* fragments were amplified by PCR using Ti042/172 (template: pUASp‐*GFP‐3FLAG‐cyp40* (Iki et al. 2020)) and Ti912/913 (template: testis cDNA), respectively, and cloned into the XbaI site of the pUASp‐K10‐attB vector by in‐Fusion reaction. For UASp‐*mTurbo‐FLAG‐ctr9t*, *mTurbo* and *FLAG‐ctr9t* fragments were amplified by PCR using Ti833/834 (template: UASp‐*mTurbo‐FLAG‐cyp40*, (Iki et al. 2023)) and Ti835/913 (template: UASp‐*GFP‐3FLAG‐ctr9t*), respectively, and cloned into the XbaI site of the pUASp‐K10‐attB vector. The constructs were integrated into the *attP2* site. For native_promoter‐*GFP‐3FLAG‐*native_3′UTR, the *GFP‐3FLAG*‐*ctr9t* fragment was amplified by PCR using eGFP‐Fw/Ti1791 (template: pUASp‐*GFP‐3FLAG‐ctr9t*), and fragments for promoter and 3′UTR regions were amplified by PCR using Ti1789/1790 and Ti1792/1793 (template: *y w* genomic DNA). The three fragments were cloned into the XbaI site of the pattB vector by in‐Fusion reaction. The construct was integrated into *attP‐3B*(*VK33*) docking site.

### 4.3 Homolog Search and Phylogenetic Analysis

Homologs of individual subunits forming Paf1C were searched by Protein BLAST (BLASTp) using the NCBI platform (https://blast.ncbi.nlm.nih.gov/Blast.cgi). Multiple sequence alignment was performed with full‐length polypeptide sequences in MAFFT version 7 (https://mafft.cbrc.jp/alignment/server/index.html) (Katoh et al. 2019). A phylogenetic tree was created by Neighbor‐Joining methods for conserved sites with the Jones‐Taylor‐Thornton (JTT) model, and visualized in phylo.io (https://phylo.io/index.html).

### 4.4 Antibody Generation

A DNA fragment for the carboxy‐terminal region (Position: 790–922) of Ctr9t was amplified by PCR using primers Ti951/952 and cloned into pENTR‐D‐Topo (Thermo Fisher Scientific). The insert was transferred to the pDEST15 destination vector by Gateway LR clonase (Thermo Fisher Scientific). GST‐tagged Ctr9t^790–922^ expressed in BL21 (DE3) was purified. Injection of the purified antigen to rabbits and guinea pigs, and serum preparation were performed by Kiwa Laboratory Animals Co. Ltd. (https://kwl‐a.co.jp/).

### 4.5 Histochemistry and Image Acquisition

Adult males were dissected in 1× PBS buffer supplemented with 0.04% (w/v) bovine serum albumin (BSA, Wako) and fixed in 5.3% (v/v) paraformaldehyde (Nacalai) in ×0.67 PBS buffer for 10 min. The samples were then incubated with 1 mM 4′,6‐Diamidine‐20‐phenylindole dihydrochloride (DAPI), Phalloidin Rhodamine × Conjugated (Wako, 1:1000) in PBX buffer (1× PBS containing 0.2%[v/v] Triton X‐100). For immunostaining, fixed testes were washed for 30 min with PBX and incubated with PBX containing 2% (w/v) BSA for blocking. The primary antibody incubation was performed overnight at 4°C, and testes were washed with PBX at 25°C for 1 h. The secondary antibody incubation was then performed at 25°C for 2 h, and testes were washed with PBX at 25°C for 1 h. The following antibodies were used in the indicated dilution: anti‐Ctr9t antibody (guinea pig, 1:5000), anti‐Ctr9t antibody (rabbit, 1:5000), anti‐Vasa antibody (guinea pig, 1:5000) (Patil and Kai 2010), anti‐Paf1 antibody (rabbit, 1:200) (Adelman et al. 2006), anti‐Cdc73 antibody (rabbit, 1:200) (Adelman et al. 2006), anti‐Fibrillarin antibody (Abcam #ab5821, rabbit, 1:500), anti‐H3K4me3 antibody (Active motif #61979, mouse, 1:100), anti‐guinea pig IgG‐Alexa Fluor 488 or 555, anti‐mouse IgG‐Alexa Fluor 488, and anti‐rabbit IgG‐Alexa Fluor 488 or 555 (Molecular probes, Invitrogen, 1:500). Images were acquired using LSM900 confocal microscope (Zeiss). Images were processed using Zen (Zeiss) and PowerPoint (Microsoft). Lengths of sperm heads in Figure 2D and intensity of Pc‐GFP signals in Figure 4E were measured using Plot_Profile in Fiji.

### 4.6 Immunoblotting

Protein samples were denatured by boiling at 95°C for 3 min in protein‐loading buffer [2% (w/v) SDS, 100 mM DTT, 0.05% (v/v) BPB, and 10% (v/v) glycerol], resolved by SDS‐PAGE, and transferred to a 0.2‐μm polyvinylidene difluoride membrane (Wako) using the semi‐dry system (Trans‐blot Turbo, Bio‐Rad). The membrane was blocked in 4% (w/v) skim milk (Nacalai) in 1× phosphate‐buffered saline (PBS) supplemented with 0.1% (v/v) Tween 20 and further incubated with the anti‐Ctr9t antibodies (1:1000; guinea pig or rabbit). Coomassie brilliant blue (CBB) staining serves as a protein loading control. Images were processed using Fiji.

### 4.7 Proximity Proteome Using TurboID

Mini(m)Turbo fusion proteins were expressed in germ cells under *bam* promoter activity using the Gal4/UASp system. After eclosion, male progenies were reared at 25°C for 3 days in the modified molasses/yeast medium supplemented with 100 μM biotin (Nacalai). Biotinylated proteins were purified from 200 testes as described (Iki et al. 2023), and resolved by SDS‐polyacrylamide gel electrophoresis (SDS‐PAGE) in a 5%–20% precast gel (ATTO). Proteins in gel particles were digested with trypsin and analyzed by liquid chromatography tandem MS (LC_MS/MS) using Q Exactive and UltiMate 3000 Nano LC (Thermo Fisher Scientific) in CoMiT Omics Center (Osaka University, Japan). The mass spectrum data were analyzed by Mascot v2.5.1 (Matrix Science).

### 4.8 Transcriptome Analysis

From 150 to 200 testes of ≤ 3‐day‐old adults, total RNAs were extracted using TRIzol LS reagent (Thermo Fisher Scientific) following the manufacturer's protocol and precipitated in the presence of 50% (v/v) isopropanol and 20 μg of glycogen (Nacalai) overnight at −20°C. After centrifugation (20,000 g, 20 min, 4C), the pellet was rinsed twice with 80% (v/v) ethanol and then resuspended in RNase‐free water. After treatment with TURBO DNase (Thermo Fisher Scientific), the RNA mixture was added with an equal volume of phenol:chloroform:isoamyl alcohol (25:24:1, pH 5.2, Nacalai), vortexed, and centrifuged (20,000 g, 3 min), and the supernatant was collected. After repeating this step, RNA in the supernatant was precipitated in the presence of 0.3 M NaOAc (pH 5.2) and 75% (v/v) ethanol overnight at −20°C. After centrifugation (20,000 g, 20 min, 4°C), the pellet was rinsed twice with 80% (v/v) ethanol, resuspended in RNase‐free water, and stored at −80°C until shipment. Library construction and sequencing were performed by Rhelixa (Japan). After enriching polyadenylated RNA using NEBNext Poly(A) mRNA Magnetic Isolation Module, libraries were made by using the NEBNext Ultra II Directional RNA Library Prep Kit. Sequencing was performed in NovaSeq 6000 (Illumina). Paired reads (150 + 150 nt) were mapped on the *D. melanogaster* genome (Drosophila_melanogaster.BDGP6.32.dna.toplevel.fa, https://metazoa.ensembl.org/) by STAR (version 2.7.10b, ‐‐outFilterMultimapNmax 100 ‐‐outSAMmultNmax 1 ‐‐outMultimapperOrder Random). Conversion to binary alignment map (bam) format and sorting were done using Samtools. Gene exon mappers were counted by featureCounts (version 2.0.1, −M ‐p ‐a dmel‐all‐r6.32.gtf (https://ftp.flybase.net/)). Using counts on exons of genes, Transcripts Per Kilobase Million (TPM) were measured. Bedgraph files were generated by bedtools genomecov (−bga ‐split ‐scale). Using the scale option, reads per million (RPM) in total exon mappers were obtained. After sorting (sort ‐k1,1 ‐k2,2n), bedgraph files between biological replicates were combined by bedtools unionbedg. Mean RPM in counting columns were given by the awk command. Final bedgraph was visualized in IGV (https://software.broadinstitute.org/software/igv/). Using the exon read counts on genes provided by featureCounts as input, the differential expression analysis was conducted by edgeR using R version 4.2.3, between control (4 replicates; 2 from *y w* and 2 from *ctr9t* ^LF/CyO^) and mutant (4 replicates; 2 from *ctr9t* ^LF/Df^ and 2 from *ctr9t* ^LF/LF^) conditions. Lowly expressed genes were filtered out with filterByExpr (12,149 genes remained, 12,133 genes had information of chromosome location). Differentially expressed genes were identified with the threshold of exact *p* < 0.01 (*n* = 286). Of note, only 56 genes were extracted when FDR < 0.05 was used as a threshold. To generate Figure 1C, raw data were collected from PRJEB22205 (Leader et al. 2018). Mapping and counting were done as described above with STAR but in a single‐end mode. Since featureCounts failed to count mappers on *Leo1*/*Atu* (probably due to the presence of overlapping noncoding RNA gene), *Leo1*/*Atu* mappers were separately counted with samtools view. To generate Figure S1B, raw data were collected from GSE99574 (Pal et al. 2023). mRNA sequences of genes encoding Paf1C subunits and the paralogs were collected from GenBank (Table S3) and used for mapping by STAR. The number of reads mapped to individual genes was counted by BBmap pileup.sh. After mapping reads to the genome by STAR, the total number of reads mapped on gene exons was obtained by featureCounts and used for TPM calculation.

### 4.9 Quantitative Reverse Transcription PCR

RNA was extracted from ~10 testes of ≤ 3‐day‐old flies for each condition using TRIzol LS reagent (Thermo Fisher Scientific) following the manufacturer's protocol. Using DNase I (NEB)–treated RNA, cDNA was synthesized with 2.5 μM oligo(dT) adaptor using Super‐Script III reverse transcriptase (Thermo Fisher Scientific). Quantitative reverse transcription PCR (qPCR) reaction was performed using KAPA SYBR FAST qPCR Master Mix (Roche) and gene‐specific primers (Table S2) in QuantStudio 5 Real‐Time PCR system (ABI). Relative transcript levels were calculated by 2^−𝛥𝛥Ct^ method using *rp49* as reference.

### 4.10 Fertility Test

Single males were mated with six *y w* virgin females for the first 3 days and with another six *y w* virgin females for the following 2 days at 25°C. The total number of hatched eggs was counted to calculate the number of progenies per day (In this male‐limited female‐saturated condition, the result can reflect the sperm production efficiency). For the fertility tests shown in Figure S2C, five females and five males aged 2–3 days were mated in single vials for 1 day (male‐saturated female‐limited condition), and the total number of hatched eggs laid within the next 2 days was counted.

## Author Contributions

Conceptualization: Taichiro Iki. Methodology: Taichiro Iki and Toshie Kai. Investigation: Taichiro Iki, Jinglan Zheng, and Toshie Kai. Supervisions: Taichiro Iki and Toshie Kai. Writing – original draft: Taichiro Iki. Writing – review and editing: Taichiro Iki and Toshie Kai.

## Conflicts of Interest

The authors declare no conflicts of interest.

## Supporting information


**Figure S1:** Phylogenetic analysis of Rtf1 homologs, and the expression pattern of Paf1C genes in different species in *Drosophila*. (A) Multiple alignment and phylogenetic analysis were done with Rtf1 protein sequences. Branch lengths measure the expected substitutions per site as indicated in the scale bar. (B) Expression patterns of genes encoding general Paf1C subunits (left) and their individual paralogs (right) in *D. yakuba*, *D. ananassae* , and *D. mojavensis* . Mean TPM ± standard deviation of triplicate data is shown. AC; abdomen without digestive or reproductive system; DG, digestive plus excretory system; f, female; GO, gonads; HD, head; m, male; RE, reproductive system without gonads; TX, thorax without digestive system; WB, whole body. Raw data are available in GSE99574.

**Figure S2:** Construction and analysis of *ctr9t* loss of function mutant. (A) CRISPR‐Cas9‐based genome editing for the generation of *ctr9t* loss of function mutant (LF mutant). Guide RNA position and homology arm regions were indicated. (B) Immunoblotting using anti‐Ctr9t antibodies raised in rabbit or guinea pig. Lysates from *ctr9t* heterozygous (+/−, LF/CyO) and homozygous mutant (−/−, LF/LF) testes were compared. Coomassie brilliant blue (CBB) staining serves as protein loading control. Asterisk indicates the nonspecific background signal. (C) Female and male fertility of *ctr9t* transheterozygous mutants (−/−, LF/Df) compared to the sibling heterozygous control (+/−, LF/CyO). *p* values: two‐tailed unpaired *t*‐test. (D) Length measurement of sperm nuclei stored in seminal vesicles. *p* values: two‐tailed unpaired *t*‐test. (E) GFP (GFP‐FLAG‐Ctr9t) signals accumulated in testes of indicated conditions. Asterisk, apical end; SC, spermatocyte; SG, spermatogonia. Arrows indicate ectopic diffuse GFP signals seen with *bam‐Gal4* and *topi‐*Gal4. With *actin5C‐Gal4*, GFP signals showed premature attenuation in spermatocytes. (F) Immunoblotting of testicular lysates using anti‐Ctr9t antibody (guinea pig). −/−, *ctr9t* ^LF/LF^; +/−, *ctr9t* ^LF/CyO^. mTurbo‐FLAG‐tagged Ctr9t was expressed in germ cells using *bam* promoter activity. CBB staining serves as protein loading control. Asterisk indicates the nonspecific background signal. (G) Proximity proteome of Ctr9t (mTurbo‐FLAG‐Ctr9t). Identified paralogs of Paf1C subunits are highlighted in gray.

**Figure S3:** Expression and localization of Ctr9t. (A) Immunostaining of Ctr9t (Green) in the presence or absence of *bam*. −/−, *Δ86/Δ86*; +/−, *Δ86/TM3*. Germ cells (Vas, red). (B) Immunostaining of general Paf1C subunit, Cdc73, in spermatocytes expressing GFP‐FLAG‐Ctr9t. (C) Immunostaining of H3K4me3 (white) and Fibrillarin (red) in spermatocytes. DNA (DAPI, blue). H3K4me3 signals overlap with autosomes but under detectable level in the nucleolar region.

**Figure S4:** Nucleoli in testes lacking *ctr9t*, Pc‐GFP localization in testes. (A) Nucleolus (Fibrillarin, red) and DNA (DAPI, blue) in the apical end of testes of *ctr9t* heterozygous control (+/−, LF/CyO) and homozygous mutant (−/−, LF/LF). (B) Testes expressing Pc‐GFP (white) immunostained for Ctr9t (red). DNA (DAPI, blue). Dotted yellow line roughly delineates the boundary between spermatogonia and spermatocytes. Nucleolar enrichment of Pc‐GFP coincided with the expression of Ctr9t. Magnified images of a spermatocyte nucleus (square box) was shown in the bottom panels.

**Figure S5:** Effect of *ctr9t* loss on testicular transcriptome. (A) Bedgraph shows RPM of known targets of tTAF (*Mst87F, dj, fzo*) in controls (*yw* and *ctr9t* ^LF^/CyO) and mutants (*ctr9t* ^LF/LF^ and *ctr9t* ^LF^/Df). Bedgraph for *ctr9t* is also provided to confirm the analyzed conditions. (B) Bedgraph shows RPM of Y chromosome‐located male fertility genes. (C) Bar graph summarizes the proportion and number of genes having positive or negative transcript log_2_FC values (*ctr9t* mutants compared to controls) on individual chromosomes. (D) Re‐analysis of whole testis and sorted spermatocyte transcriptome data (available as GSE263955). Results of differential expression analysis (indicated mutant conditions compared to *w1118* wild‐type control) were summarized as bar graphs, similar to Panel C. (E) Information for the list of 33 genes expressed from Y chromosome (those given log_2_FC values after differential expression analysis). (F) RT‐qPCR measurement of transcript levels for selected genes on sex chromosomes. Four biological replicates. *p* values; two‐tailed unpaired *t*‐test.

**Table S1:** Summary of transcriptome analyses.

**Table S2:** Oligonucleotides used in this study.

**Table S3:** List of genes encoding Paf1C subunits and the paralogs in *D. yakuba D. ananasseae D. mojavensis*.

## Data Availability

The data that support the findings of this study are openly available in NIH at https://www.ncbi.nlm.nih.gov/bioproject/PRJNA1094673/, reference number PRJNA1094673.
