# Supplementary material for: Paralog‐Dependent Specialization of Paf1C Subunit, Ctr9, for Sex Chromosome Gene Regulation and Male Germline Differentiation in Drosophila
Source: Genes Cells. 2025 Aug 5;30(5):e70040. doi: 10.1111/gtc.70040 (PMC12324932; doi:10.1111/gtc.70040)
Supplement: Supplementary file 1 — Figure S1: Phylogenetic analysis of Rtf1 homologs, and the expression pattern of Paf1C genes in different species in Drosophila. (A) Multiple alignment and phylogenetic analysis were done with Rtf1 protein sequences. Branch lengths measure the expected substitutions per site as indicated in the scale bar. (B) Expression patterns of genes encoding general Paf1C subunits (left) and their individual paralogs (right) in D. yakuba, D. ananassae , and D. mojavensis . Mean TPM ± standard deviation of triplicate data is shown. AC; abdomen without digestive or reproductive system; DG, digestive plus excretory system; f, female; GO, gonads; HD, head; m, male; RE, reproductive system without gonads; TX, thorax without digestive system; WB, whole body. Raw data are available in GSE99574. [file GTC-30-0-s002.pptx]

## Slide 1
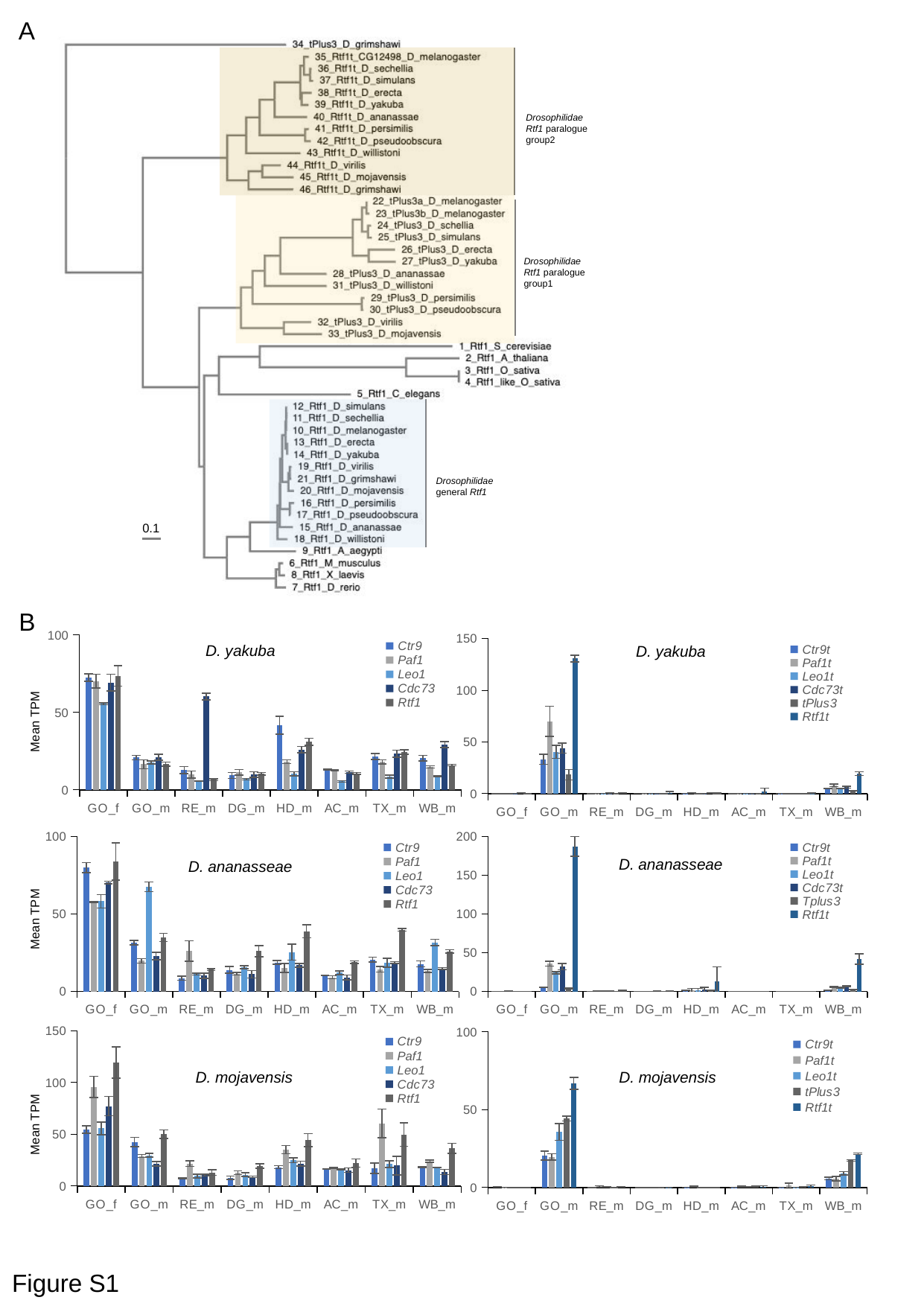

A
Drosophilidae
Rtf1 paralogue
group2
Drosophilidae
Rtf1 paralogue
group1
Drosophilidae
general Rtf1
0.1
B
### Chart
| Category | Ctr9 | Paf1 | Leo1 | Cdc73 | Rtf1 |
|---|---|---|---|---|---|
| GO_f | 72.46994661058143 | 70.27299530637426 | 55.67342615591388 | 69.26314848603253 | 73.58926205234205 |
| GO_m | 20.854828073011035 | 16.562256926244476 | 17.788979720765955 | 21.124440940223156 | 16.68374291110837 |
| RE_m | 12.851643578623387 | 9.944982534009283 | 5.732527542182076 | 60.30337367421149 | 6.503143983020433 |
| DG_m | 9.330116140364954 | 11.33916360845061 | 6.958775307727983 | 9.98118729386289 | 10.38087690092315 |
| HD_m | 41.8178270063608 | 18.19057665806972 | 10.391095899301341 | 26.103509741353033 | 31.127484255269383 |
| AC_m | 13.268313279046774 | 12.847057010853623 | 5.573369538222598 | 11.485422633664408 | 10.594793492534068 |
| TX_m | 21.56154871667522 | 18.037332415495353 | 8.61940415312088 | 23.36365220564673 | 24.47446503670533 |
| WB_m | 20.460645934657947 | 14.785071970391067 | 8.781409108917197 | 29.299252021018628 | 16.01441806355886 |
### Chart
| Category | Ctr9t | Paf1t | Leo1t | Cdc73t | tPlus3 | Rtf1t |
|---|---|---|---|---|---|---|
| GO_f | 0.0 | 0.0 | 0.0 | 0.046430089284885505 | 0.5116869866895476 | 0.0 |
| GO_m | 33.33904465500073 | 69.86982700888134 | 40.48786906753369 | 43.95240592463748 | 18.226570672962485 | 130.8298145718961 |
| RE_m | 0.015411190371730487 | 0.12216963465616239 | 0.039342488384154405 | 0.19805545859252024 | 0.024584152138353712 | 0.26966967204119746 |
| DG_m | 0.22342289176514105 | 0.11022450562807005 | 0.053243668255122785 | 0.0893452987386833 | 0.06528632249100759 | 0.8690226322629743 |
| HD_m | 0.1218263918651496 | 0.3299573120146512 | 0.12972699029270018 | 0.04976756371214411 | 0.22239104741558313 | 0.2572662753480459 |
| AC_m | 0.14434977484053937 | 0.12986889419654943 | 0.09409926059355085 | 0.048984454075076796 | 0.1905645040300905 | 2.3083983850864 |
| TX_m | 0.04438351803171811 | 0.0 | 0.0 | 0.0 | 0.0 | 0.16208509793743361 |
| WB_m | 5.011254067692354 | 7.952099854957349 | 4.9329811306652696 | 6.1359807020606825 | 2.636786218177674 | 19.420876089197606 |D. yakuba
D. yakuba
Mean TPM
### Chart
| Category | Ctr9t | Paf1t | Leo1t | Cdc73t | Tplus3 | Rtf1t |
|---|---|---|---|---|---|---|
| GO_f | 0.025961767286639553 | 0.0 | 0.10766534840230146 | 0.0 | 0.0 | 0.0 |
| GO_m | 5.279751913179584 | 35.73023006516461 | 24.04693646331121 | 32.156840844911756 | 4.255271529885808 | 187.19512349581046 |
| RE_m | 0.0 | 0.11878951453200566 | 0.1506886451606492 | 0.2206335009862003 | 0.032431990951186575 | 1.0262401645922512 |
| DG_m | 0.0 | 0.0 | 0.0 | 0.1202822261849291 | 0.0 | 0.09965514011369568 |
| HD_m | 0.6717118690445543 | 1.648159524542997 | 1.671206688357887 | 2.1152850271410295 | 0.6164796989268094 | 13.266712953647291 |
| AC_m | 0.0 | 0.0 | 0.0 | 0.04138384886860752 | 0.0 | 0.09112459431471091 |
| TX_m | 0.0 | 0.0 | 0.022086767496197927 | 0.0652067599163731 | 0.0 | 0.081036770792331 |
| WB_m | 1.4785902487784128 | 5.405153264778169 | 4.758821159029672 | 6.355175546430744 | 2.12340182311802 | 41.695021308547986 |
### Chart
| Category | Ctr9 | Paf1 | Leo1 | Cdc73 | Rtf1 |
|---|---|---|---|---|---|
| GO_f | 79.79385539372323 | 57.58910233604136 | 58.1845102867959 | 70.33088168397002 | 83.87192813537666 |
| GO_m | 31.537200185825828 | 19.711090371365668 | 67.53634113403582 | 22.821372825507922 | 34.8210155816382 |
| RE_m | 8.480544850971683 | 26.04631314902731 | 11.13386588681953 | 10.152281039434257 | 14.106637715747738 |
| DG_m | 13.912955484130704 | 11.328511076183384 | 15.66152325622867 | 11.094243512696403 | 25.975186436737783 |
| HD_m | 18.619875195634 | 15.028458091225682 | 25.36603213365991 | 16.797354329709993 | 38.795963538790424 |
| AC_m | 10.459656454741971 | 9.13112607348461 | 12.06313772108846 | 8.99158166116083 | 18.878858362993245 |
| TX_m | 20.478085670638418 | 14.367206602583957 | 18.518620316174758 | 18.54261281572691 | 39.75115125300701 |
| WB_m | 17.666043449874056 | 13.271478854439266 | 31.602454193580797 | 14.812209561108743 | 25.765192309213834 |D. ananasseae
D. ananasseae
Mean TPM
### Chart
| Category | Ctr9 | Paf1 | Leo1 | Cdc73 | Rtf1 |
|---|---|---|---|---|---|
| GO_f | 54.631762800364875 | 95.67713017164412 | 55.6762323460274 | 77.1320295527509 | 119.25428530992714 |
| GO_m | 42.34630042048972 | 29.00024274093442 | 29.564163383563358 | 21.395239717719917 | 50.025649685799614 |
| RE_m | 7.188514088056355 | 21.608061994533255 | 9.652997505786312 | 10.635635448493609 | 12.989240374896278 |
| DG_m | 8.06404882524584 | 12.835304756165621 | 11.016988997445877 | 8.807116306175638 | 19.443783006832287 |
| HD_m | 18.18780143683081 | 35.101846385650354 | 24.968849328229663 | 21.497513187326096 | 44.32124510586223 |
| AC_m | 16.537828397150125 | 17.365496148470992 | 15.786683081453509 | 15.325925616454546 | 22.10452493522712 |
| TX_m | 17.207947628259237 | 60.35050539767375 | 21.13339196834094 | 19.74433963197007 | 49.72073679978447 |
| WB_m | 18.2444330228487 | 23.897272145699542 | 17.721155401589673 | 13.57805276899154 | 36.37313279384477 |
### Chart
| Category | Ctr9t | Paf1t | Leo1t | tPlus3 | Rtf1t |
|---|---|---|---|---|---|
| GO_f | 0.16223480261266707 | 0.08727325372595929 | 0.0 | 0.0 | 0.0 |
| GO_m | 20.479053420637566 | 19.59434918318298 | 35.67941845885501 | 44.35304192676415 | 66.89897558167443 |
| RE_m | 0.0 | 0.6077572333113668 | 0.12577919685049918 | 0.0 | 0.3052786921138541 |
| DG_m | 0.0 | 0.0 | 0.0 | 0.08624629209708634 | 0.0832651722424514 |
| HD_m | 0.025364738981212696 | 0.4365378654747514 | 0.0 | 0.0 | 0.0 |
| AC_m | 0.07401107998655651 | 0.7072126276904341 | 0.15059443640040626 | 0.4621120003422168 | 0.4575151043518755 |
| TX_m | 0.01663235331062317 | 1.3829181511443804 | 0.08902369999167942 | 0.2525126983881366 | 0.7031764459079838 |
| WB_m | 5.716339748465256 | 5.657948026305522 | 9.105718819688823 | 17.415294544640684 | 21.643283328573066 |D. mojavensis
D. mojavensis
Mean TPM
Figure S1
