# Supplementary material for: Paralog‐Dependent Specialization of Paf1C Subunit, Ctr9, for Sex Chromosome Gene Regulation and Male Germline Differentiation in Drosophila
Source: Genes Cells. 2025 Aug 5;30(5):e70040. doi: 10.1111/gtc.70040 (PMC12324932; doi:10.1111/gtc.70040)
Supplement: Supplementary file 2 — Figure S2: Construction and analysis of ctr9t loss of function mutant. (A) CRISPR‐Cas9‐based genome editing for the generation of ctr9t loss of function mutant (LF mutant). Guide RNA position and homology arm regions were indicated. (B) Immunoblotting using anti‐Ctr9t antibodies raised in rabbit or guinea pig. Lysates from ctr9t heterozygous (+/−, LF/CyO) and homozygous mutant (−/−, LF/LF) testes were compared. Coomassie brilliant blue (CBB) staining serves as protein loading control. Asterisk indicates the nonspecific background signal. (C) Female and male fertility of ctr9t transheterozygous mutants (−/−, LF/Df) compared to the sibling heterozygous control (+/−, LF/CyO). p values: two‐tailed unpaired t‐test. (D) Length measurement of sperm nuclei stored in seminal vesicles. p values: two‐tailed unpaired t‐test. (E) GFP (GFP‐FLAG‐Ctr9t) signals accumulated in testes of indicated conditions. Asterisk, apical end; SC, spermatocyte; SG, spermatogonia. Arrows indicate ectopic diffuse GFP signals seen with bam‐Gal4 and topi‐Gal4. With actin5C‐Gal4, GFP signals showed premature attenuation in spermatocytes. (F) Immunoblotting of testicular lysates using anti‐Ctr9t antibody (guinea pig). −/−, ctr9t LF/LF; +/−, ctr9t LF/CyO. mTurbo‐FLAG‐tagged Ctr9t was expressed in germ cells using bam promoter activity. CBB staining serves as protein loading control. Asterisk indicates the nonspecific background signal. (G) Proximity proteome of Ctr9t (mTurbo‐FLAG‐Ctr9t). Identified paralogs of Paf1C subunits are highlighted in gray. [file GTC-30-0-s004.pptx]

## Slide 1
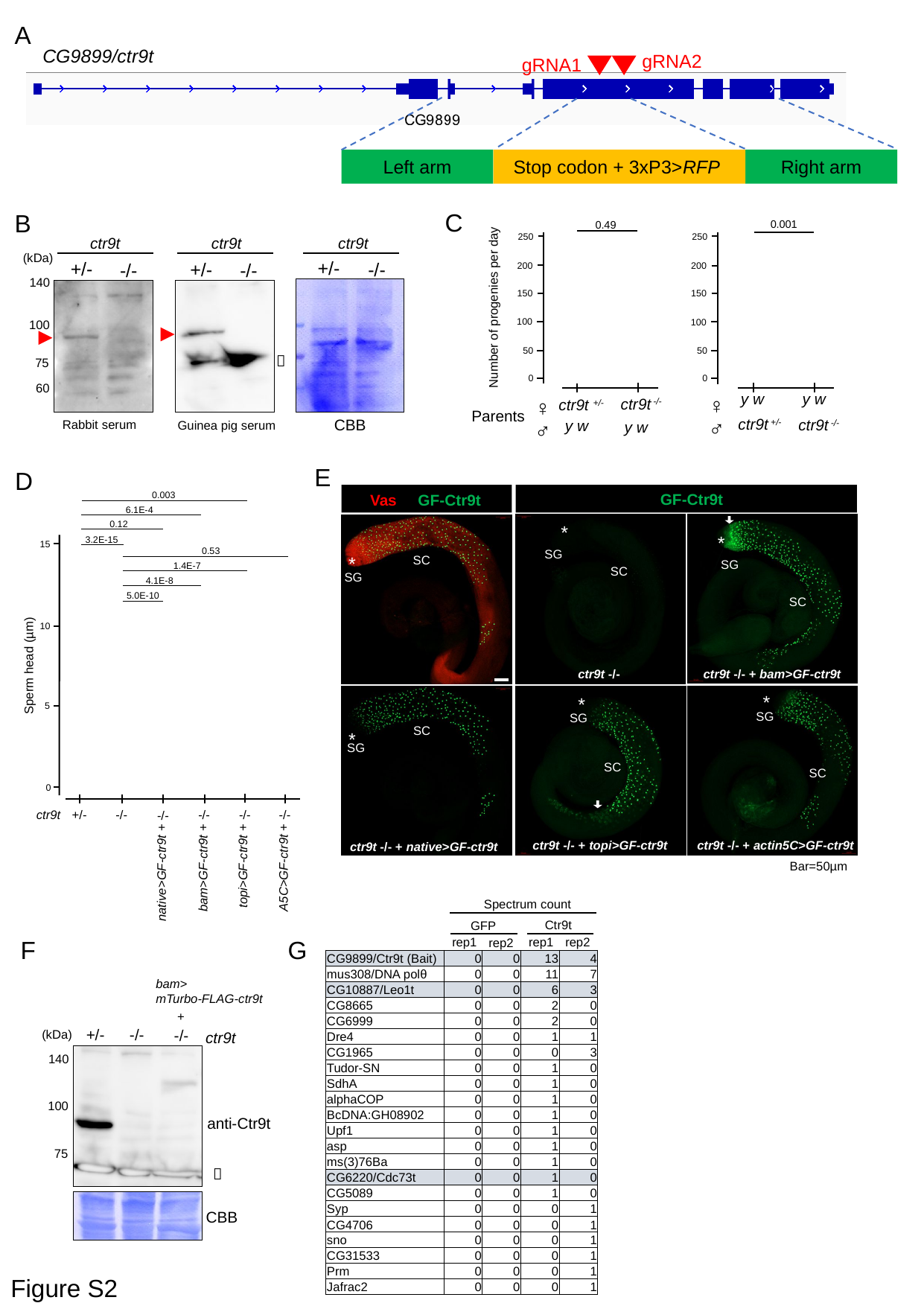

A
CG9899/ctr9t
gRNA2
gRNA1
Left arm
Right arm
Stop codon + 3xP3>RFP
C
B
0.001
0.49
250
200
150
100
50
0
250
200
150
100
50
0
ctr9t
ctr9t
ctr9t
(kDa)
+/-
+/-
-/-
+/-
-/-
-/-
140
Number of progenies per day
100
＊
75
60
y w
y w
♀
♀
ctr9t -/-
ctr9t +/-
Parents
♂
ctr9t +/-
ctr9t -/-
CBB
y w
Rabbit serum
♂
y w
Guinea pig serum
E
D
0.003
GF-Ctr9t
GF-Ctr9t
Vas
6.1E-4
0.12
*
*
3.2E-15
15
0.53
SG
*
SC
SG
1.4E-7
SC
SG
4.1E-8
5.0E-10
SC
10
Sperm head (µm)
ctr9t -/-
ctr9t -/- + bam>GF-ctr9t
*
*
5
SG
SG
SC
*
SG
SC
SC
0
ctr9t
+/-
-/-
-/-
-/-
-/-
-/-
ctr9t -/- + topi>GF-ctr9t
ctr9t -/- + actin5C>GF-ctr9t
ctr9t -/- + native>GF-ctr9t
bam>GF-ctr9t +
topi>GF-ctr9t +
A5C>GF-ctr9t +
Bar=50µm
native>GF-ctr9t +
Spectrum count
Ctr9t
GFP
rep1
rep1
rep2
rep2
F
G
| CG9899/Ctr9t (Bait) | 0 | 0 | 13 | 4 |
| --- | --- | --- | --- | --- |
| mus308/DNA polθ | 0 | 0 | 11 | 7 |
| CG10887/Leo1t | 0 | 0 | 6 | 3 |
| CG8665 | 0 | 0 | 2 | 0 |
| CG6999 | 0 | 0 | 2 | 0 |
| Dre4 | 0 | 0 | 1 | 1 |
| CG1965 | 0 | 0 | 0 | 3 |
| Tudor-SN | 0 | 0 | 1 | 0 |
| SdhA | 0 | 0 | 1 | 0 |
| alphaCOP | 0 | 0 | 1 | 0 |
| BcDNA:GH08902 | 0 | 0 | 1 | 0 |
| Upf1 | 0 | 0 | 1 | 0 |
| asp | 0 | 0 | 1 | 0 |
| ms(3)76Ba | 0 | 0 | 1 | 0 |
| CG6220/Cdc73t | 0 | 0 | 1 | 0 |
| CG5089 | 0 | 0 | 1 | 0 |
| Syp | 0 | 0 | 0 | 1 |
| CG4706 | 0 | 0 | 0 | 1 |
| sno | 0 | 0 | 0 | 1 |
| CG31533 | 0 | 0 | 0 | 1 |
| Prm | 0 | 0 | 0 | 1 |
| Jafrac2 | 0 | 0 | 0 | 1 |
bam>
mTurbo-FLAG-ctr9t
+
+/-
-/-
-/-
(kDa)
ctr9t
140
100
anti-Ctr9t
75
＊
CBB
Figure S2
