# Supplementary material for: Paralog‐Dependent Specialization of Paf1C Subunit, Ctr9, for Sex Chromosome Gene Regulation and Male Germline Differentiation in Drosophila
Source: Genes Cells. 2025 Aug 5;30(5):e70040. doi: 10.1111/gtc.70040 (PMC12324932; doi:10.1111/gtc.70040)
Supplement: Supplementary file 3 — Figure S3: Expression and localization of Ctr9t. (A) Immunostaining of Ctr9t (Green) in the presence or absence of bam. −/−, Δ86/Δ86; +/−, Δ86/TM3. Germ cells (Vas, red). (B) Immunostaining of general Paf1C subunit, Cdc73, in spermatocytes expressing GFP‐FLAG‐Ctr9t. (C) Immunostaining of H3K4me3 (white) and Fibrillarin (red) in spermatocytes. DNA (DAPI, blue). H3K4me3 signals overlap with autosomes but under detectable level in the nucleolar region. [file GTC-30-0-s005.pptx]

## Slide 1
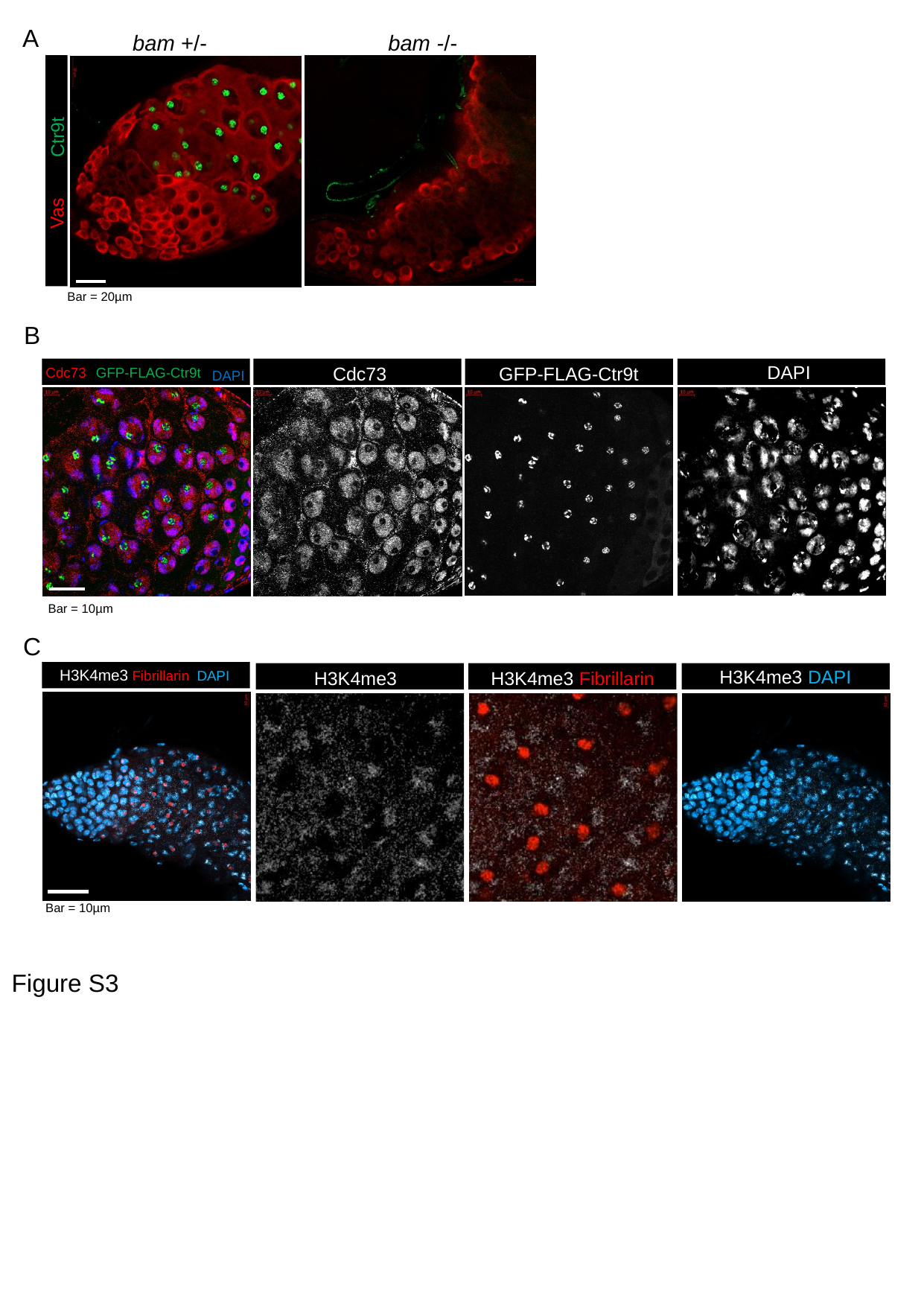

A
bam +/-
bam -/-
Vas
Ctr9t
Bar = 20µm
B
DAPI
GFP-FLAG-Ctr9t
Cdc73
Cdc73
GFP-FLAG-Ctr9t
DAPI
Bar = 10µm
C
H3K4me3 Fibrillarin DAPI
H3K4me3 DAPI
H3K4me3
H3K4me3 Fibrillarin
Bar = 10µm
Figure S3
