# Supplementary figures and images for: Paralog‐Dependent Specialization of Paf1C Subunit, Ctr9, for Sex Chromosome Gene Regulation and Male Germline Differentiation in Drosophila
Source: Genes Cells. 2025 Aug 5;30(5):e70040. doi: 10.1111/gtc.70040 (PMC12324932; doi:10.1111/gtc.70040)

## Slide 1
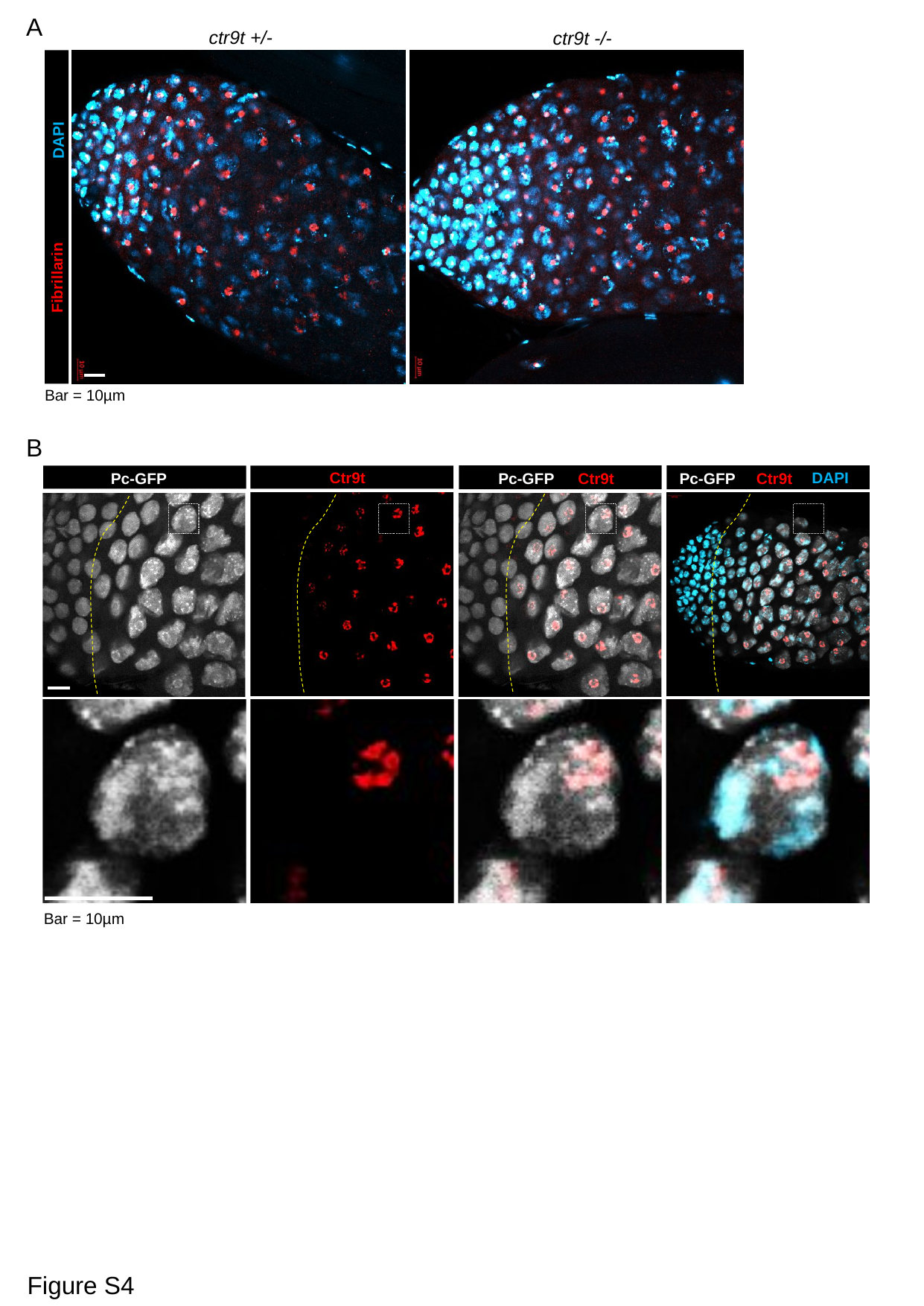

A
ctr9t +/-
ctr9t -/-
DAPI
Fibrillarin
Bar = 10µm
B
Ctr9t
DAPI
Pc-GFP
Pc-GFP
Ctr9t
Pc-GFP
Ctr9t
Bar = 10µm
Figure S4

Supplement: Supplementary file 4 — Figure S4: Nucleoli in testes lacking ctr9t, Pc‐GFP localization in testes. (A) Nucleolus (Fibrillarin, red) and DNA (DAPI, blue) in the apical end of testes of ctr9t heterozygous control (+/−, LF/CyO) and homozygous mutant (−/−, LF/LF). (B) Testes expressing Pc‐GFP (white) immunostained for Ctr9t (red). DNA (DAPI, blue). Dotted yellow line roughly delineates the boundary between spermatogonia and spermatocytes. Nucleolar enrichment of Pc‐GFP coincided with the expression of Ctr9t. Magnified images of a spermatocyte nucleus (square box) was shown in the bottom panels. [file GTC-30-0-s001.pptx]
