# Supplementary material for: Paralog‐Dependent Specialization of Paf1C Subunit, Ctr9, for Sex Chromosome Gene Regulation and Male Germline Differentiation in Drosophila
Source: Genes Cells. 2025 Aug 5;30(5):e70040. doi: 10.1111/gtc.70040 (PMC12324932; doi:10.1111/gtc.70040)
Supplement: Supplementary file 5 — Figure S5: Effect of ctr9t loss on testicular transcriptome. (A) Bedgraph shows RPM of known targets of tTAF (Mst87F, dj, fzo) in controls (yw and ctr9t LF/CyO) and mutants (ctr9t LF/LF and ctr9t LF/Df). Bedgraph for ctr9t is also provided to confirm the analyzed conditions. (B) Bedgraph shows RPM of Y chromosome‐located male fertility genes. (C) Bar graph summarizes the proportion and number of genes having positive or negative transcript log2FC values (ctr9t mutants compared to controls) on individual chromosomes. (D) Re‐analysis of whole testis and sorted spermatocyte transcriptome data (available as GSE263955). Results of differential expression analysis (indicated mutant conditions compared to w1118 wild‐type control) were summarized as bar graphs, similar to Panel C. (E) Information for the list of 33 genes expressed from Y chromosome (those given log2FC values after differential expression analysis). (F) RT‐qPCR measurement of transcript levels for selected genes on sex chromosomes. Four biological replicates. p values; two‐tailed unpaired t‐test. [file GTC-30-0-s003.pptx]

## Slide 1
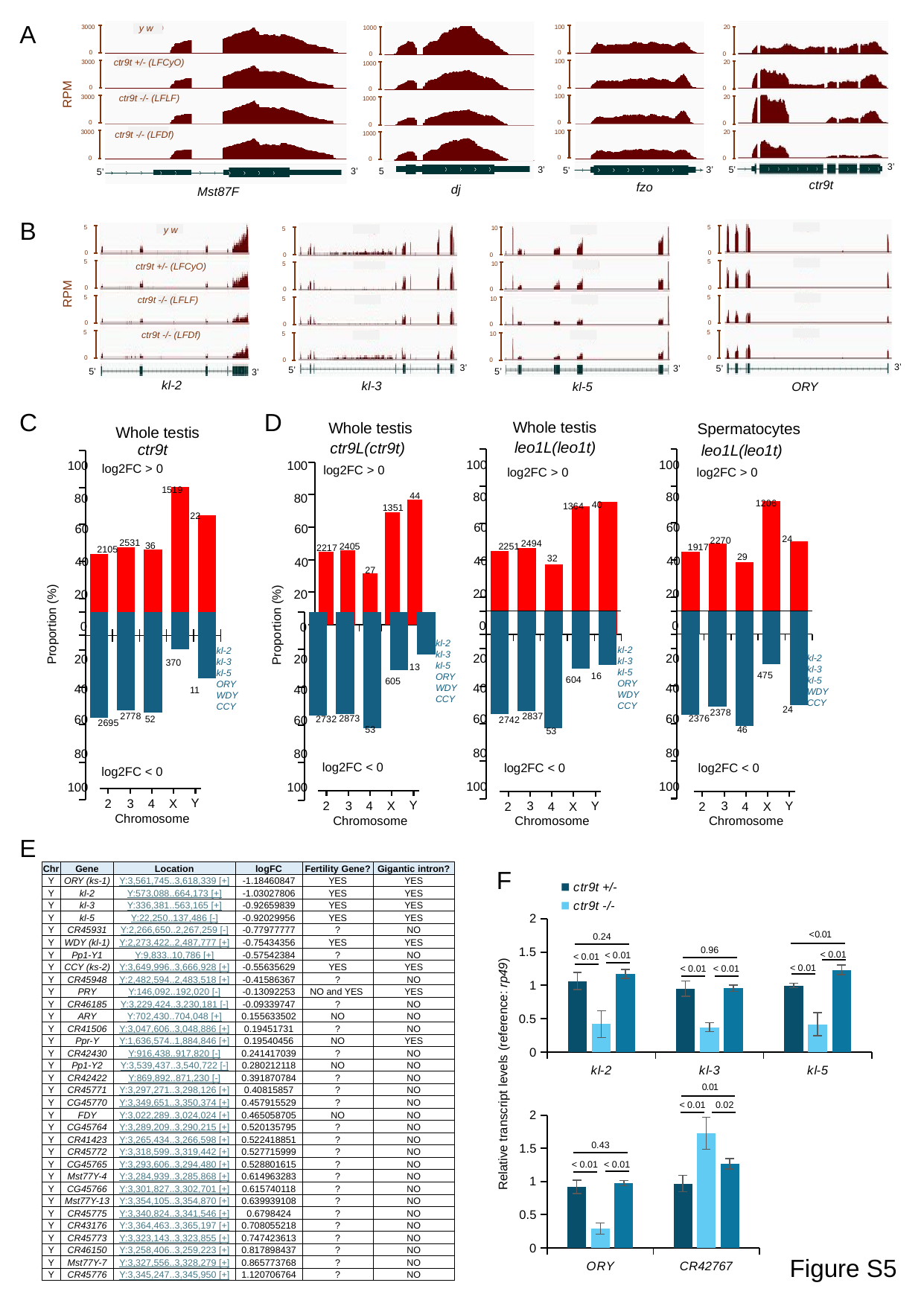

A
y w
3000
0
100
0
20
0
1000
0
ctr9t +/- (LFCyO)
100
0
3000
0
20
0
1000
0
RPM
ctr9t -/- (LFLF)
100
0
3000
0
20
0
1000
0
20
0
100
0
3000
0
ctr9t -/- (LFDf)
1000
0
3’
3’
3’
5’
5’
5’
3’
5’
ctr9t
fzo
dj
Mst87F
B
5
0
5
0
y w
10
0
5
0
5
0
5
0
5
0
10
0
ctr9t +/- (LFCyO)
RPM
5
0
5
0
ctr9t -/- (LFLF)
5
0
10
0
5
0
5
0
ctr9t -/- (LFDf)
5
0
10
0
3’
3’
5’
3’
5’
5’
5’
3’
kl-2
kl-3
kl-5
ORY
C
D
Whole testis
Whole testis
Spermatocytes
Whole testis
leo1L(leo1t)
ctr9L(ctr9t)
ctr9t
leo1L(leo1t)
### Chart
| Category | Up |
|---|---|
| 2 | 44.65408805031446 |
| 3 | 48.83820998278829 |
| 4 | 38.666666666666664 |
| X | 71.74301011302796 |
| Y | 50.0 |
### Chart
| Category | Up |
|---|---|
| 2 | 44.79692867245909 |
| 3 | 45.566502463054185 |
| 4 | 31.3953488372093 |
| X | 69.06952965235173 |
| Y | 77.19298245614034 |
### Chart
| Category | Up |
|---|---|
| 2 | 45.08311636290807 |
| 3 | 46.78296754830239 |
| 4 | 37.64705882352941 |
| X | 69.3089430894309 |
| Y | 71.42857142857143 |
### Chart
| Category | Up |
|---|---|
| 2 | 43.854166666666664 |
| 3 | 47.664783427495294 |
| 4 | 46.391752577319586 |
| X | 80.37037037037037 |
| Y | 64.70588235294117 |100
80
60
40
20
0
20
40
60
80
100
100
80
60
40
20
0
20
40
60
80
100
100
100
log2FC > 0
log2FC > 0
log2FC > 0
log2FC > 0
1519
44
80
80
1206
40
1364
1351
22
60
60
24
2270
2531
2494
36
2405
2251
1917
2217
2105
29
32
40
40
27
20
20
### Chart
| Category | Down |
|---|---|
| 2 | -55.3459119496855 |
| 3 | -51.1617900172117 |
| 4 | -61.3333333333333 |
| X | -28.256989886972 |
| Y | -50.0 |
### Chart
| Category | Down |
|---|---|
| 2 | -54.9168836370919 |
| 3 | -53.2170324516976 |
| 4 | -62.3529411764706 |
| X | -30.6910569105691 |
| Y | -28.5714285714286 |
### Chart
| Category | Down |
|---|---|
| 2 | -55.2030713275409 |
| 3 | -54.4334975369458 |
| 4 | -61.6279069767442 |
| X | -30.9304703476483 |
| Y | -22.8070175438596 |
### Chart
| Category | Down |
|---|---|
| 2 | -56.1458333333333 |
| 3 | -52.3352165725047 |
| 4 | -53.6082474226804 |
| X | -19.6296296296296 |
| Y | -35.2941176470588 |Proportion (%)
Proportion (%)
0
0
kl-2
kl-3
kl-5
ORY
WDY
CCY
kl-2
kl-3
kl-5
ORY
WDY
CCY
kl-2
kl-3
kl-5
ORY
WDY
CCY
20
20
kl-2
kl-3
kl-5
WDY
CCY
370
13
475
16
604
605
40
40
11
24
2378
2837
2778
60
60
2376
2873
2732
52
2742
2695
46
53
53
80
80
log2FC < 0
log2FC < 0
Y
3
2
X
4
Chromosome
log2FC < 0
Y
3
2
X
4
Chromosome
log2FC < 0
100
100
Y
3
2
X
Y
3
2
X
4
Chromosome
4
Chromosome
### Chart
| Category | ctr9t +/- | ctr9t -/- | ctr9t -/- + native promoter>GFP-FLAG-ctr9t |
|---|---|---|---|
| kl-2 | 1.0636837184429169 | 0.4179118201136589 | 1.170923799276352 |
| kl-3 | 0.9520312398672104 | 0.37079451233148575 | 0.9556577801704407 |
| kl-5 | 0.997365266084671 | 0.4172479547560215 | 1.231964260339737 |E
F
| Chr | Gene | Location | logFC | Fertility Gene? | Gigantic intron? |
| --- | --- | --- | --- | --- | --- |
| Y | ORY (ks-1) | Y:3,561,745..3,618,339 [+] | -1.18460847 | YES | YES |
| Y | kl-2 | Y:573,088..664,173 [+] | -1.03027806 | YES | YES |
| Y | kl-3 | Y:336,381..563,165 [+] | -0.92659839 | YES | YES |
| Y | kl-5 | Y:22,250..137,486 [-] | -0.92029956 | YES | YES |
| Y | CR45931 | Y:2,266,650..2,267,259 [-] | -0.77977777 | ? | NO |
| Y | WDY (kl-1) | Y:2,273,422..2,487,777 [+] | -0.75434356 | YES | YES |
| Y | Pp1-Y1 | Y:9,833..10,786 [+] | -0.57542384 | ? | NO |
| Y | CCY (ks-2) | Y:3,649,996..3,666,928 [+] | -0.55635629 | YES | YES |
| Y | CR45948 | Y:2,482,594..2,483,518 [+] | -0.41586367 | ? | NO |
| Y | PRY | Y:146,092..192,020 [-] | -0.13092253 | NO and YES | YES |
| Y | CR46185 | Y:3,229,424..3,230,181 [-] | -0.09339747 | ? | NO |
| Y | ARY | Y:702,430..704,048 [+] | 0.155633502 | NO | NO |
| Y | CR41506 | Y:3,047,606..3,048,886 [+] | 0.19451731 | ? | NO |
| Y | Ppr-Y | Y:1,636,574..1,884,846 [+] | 0.19540456 | NO | YES |
| Y | CR42430 | Y:916,438..917,820 [-] | 0.241417039 | ? | NO |
| Y | Pp1-Y2 | Y:3,539,437..3,540,722 [-] | 0.280212118 | NO | NO |
| Y | CR42422 | Y:869,892..871,230 [-] | 0.391870784 | ? | NO |
| Y | CR45771 | Y:3,297,271..3,298,126 [+] | 0.40815857 | ? | NO |
| Y | CG45770 | Y:3,349,651..3,350,374 [+] | 0.457915529 | ? | NO |
| Y | FDY | Y:3,022,289..3,024,024 [+] | 0.465058705 | NO | NO |
| Y | CG45764 | Y:3,289,209..3,290,215 [+] | 0.520135795 | ? | NO |
| Y | CR41423 | Y:3,265,434..3,266,598 [+] | 0.522418851 | ? | NO |
| Y | CR45772 | Y:3,318,599..3,319,442 [+] | 0.527715999 | ? | NO |
| Y | CG45765 | Y:3,293,606..3,294,480 [+] | 0.528801615 | ? | NO |
| Y | Mst77Y-4 | Y:3,284,939..3,285,868 [+] | 0.614963283 | ? | NO |
| Y | CG45766 | Y:3,301,827..3,302,701 [+] | 0.615740118 | ? | NO |
| Y | Mst77Y-13 | Y:3,354,105..3,354,870 [+] | 0.639939108 | ? | NO |
| Y | CR45775 | Y:3,340,824..3,341,546 [+] | 0.6798424 | ? | NO |
| Y | CR43176 | Y:3,364,463..3,365,197 [+] | 0.708055218 | ? | NO |
| Y | CR45773 | Y:3,323,143..3,323,855 [+] | 0.747423613 | ? | NO |
| Y | CR46150 | Y:3,258,406..3,259,223 [+] | 0.817898437 | ? | NO |
| Y | Mst77Y-7 | Y:3,327,556..3,328,279 [+] | 0.865773768 | ? | NO |
| Y | CR45776 | Y:3,345,247..3,345,950 [+] | 1.120706764 | ? | NO |
0.24
0.96
< 0.01
< 0.01
< 0.01
< 0.01
< 0.01
< 0.01
Relative transcript levels (reference: rp49)
### Chart
| Category | ctr9t +/- | ctr9t -/- | ctr9t -/- + native promoter>GFP-FLAG-ctr9t |
|---|---|---|---|
| ORY | 0.9200198799371719 | 0.2902778498828411 | 0.9729558676481247 |
| CR42767 | 0.9670521169900894 | 1.7251662015914917 | 1.267973929643631 |0.02
< 0.01
0.43
< 0.01
< 0.01
Figure S5
